# Unravelling infiltrating T‐cell heterogeneity in kidney renal clear cell carcinoma: Integrative single‐cell and spatial transcriptomic profiling

**DOI:** 10.1111/jcmm.18403

**Published:** 2024-06-21

**Authors:** Haiqing Chen, Haoyuan Zuo, Jinbang Huang, Jie Liu, Lai Jiang, Chenglu Jiang, Shengke Zhang, Qingwen Hu, Haotian Lai, Bangchao Yin, Guanhu Yang, Gang Mai, Bo Li, Hao Chi

**Affiliations:** ^1^ Department of General Surgery (Hepatopancreatobiliary Surgery), The Affiliated Hospital Southwest Medical University Luzhou China; ^2^ School of Clinical Medicine, The Affiliated Hospital Southwest Medical University Luzhou China; ^3^ Department of General Surgery (Hepatopancreatobiliary Surgery) Deyang People's Hospital Deyang China; ^4^ Department of General Surgery Dazhou Central Hospital Dazhou China; ^5^ Department of Pathology Sixth People's Hospital of Yibin Yibin China; ^6^ Department of Specialty Medicine Ohio University Athens Ohio USA

**Keywords:** immune microenvironment, machine learning, multi‐omics, single‐cell analysis, spatial transcriptome, T cells, tumour heterogeneity

## Abstract

Kidney renal clear cell carcinoma (KIRC) pathogenesis intricately involves immune system dynamics, particularly the role of T cells within the tumour microenvironment. Through a multifaceted approach encompassing single‐cell RNA sequencing, spatial transcriptome analysis and bulk transcriptome profiling, we systematically explored the contribution of infiltrating T cells to KIRC heterogeneity. Employing high‐density weighted gene co‐expression network analysis (hdWGCNA), module scoring and machine learning, we identified a distinct signature of infiltrating T cell‐associated genes (ITSGs). Spatial transcriptomic data were analysed using robust cell type decomposition (RCTD) to uncover spatial interactions. Further analyses included enrichment assessments, immune infiltration evaluations and drug susceptibility predictions. Experimental validation involved PCR experiments, CCK‐8 assays, plate cloning assays, wound‐healing assays and Transwell assays. Six subpopulations of infiltrating and proliferating T cells were identified in KIRC, with notable dynamics observed in mid‐ to late‐stage disease progression. Spatial analysis revealed significant correlations between T cells and epithelial cells across varying distances within the tumour microenvironment. The ITSG‐based prognostic model demonstrated robust predictive capabilities, implicating these genes in immune modulation and metabolic pathways and offering prognostic insights into drug sensitivity for 12 KIRC treatment agents. Experimental validation underscored the functional relevance of PPIB in KIRC cell proliferation, invasion and migration. Our study comprehensively characterizes infiltrating T‐cell heterogeneity in KIRC using single‐cell RNA sequencing and spatial transcriptome data. The stable prognostic model based on ITSGs unveils infiltrating T cells' prognostic potential, shedding light on the immune microenvironment and offering avenues for personalized treatment and immunotherapy.

## 1 INTRODUCTION

Renal cancer, a prevalent malignancy in humans, has garnered considerable attention due to its elevated morbidity and mortality rate.^1^, ^2^ Kidney renal clear cell carcinoma (KIRC), representing a predominant subset within renal cell carcinoma, comprises a significant proportion of incidences in renal cancer. Diverging from other subtypes, KIRC commonly presents with minimal symptoms during its initial stages, thereby contributing to its predilection for late‐stage diagnoses.^3^, ^4^ Despite diverse treatment modalities, advanced KIRC exhibits marked resistance to conventional chemotherapy and radiotherapy.^5^, ^6^, ^7^ Recent advancements in bioinformatics have catalysed the emergence of precision medicine, affording a more nuanced foundation for personalized treatment approaches.^8^ Consequently, a comprehensive comprehension of the pathophysiological mechanisms underlying renal clear cell carcinoma, coupled with the identification of biomarkers and their integration with effective immunotherapeutic strategies, constitutes pivotal facets of contemporary research. Precision medicine, when applied to renal clear cell carcinoma, signifies a tailored and efficacious treatment paradigm, with the potential to enhance both patient survival and quality of life. The progression of renal clear cell carcinoma (RCCC) is intricately linked to the immune system, with a particular emphasis on the distribution and function of T cells within the tumour microenvironment, exerting a direct influence on RCCC's growth and dissemination.^9^, ^10^, ^11^ However, the heterogeneity within these T‐cell populations remains a subject warranting more comprehensive exploration.

The intricate immune microenvironment comprises a network that includes immune cells, extracellular matrix and diverse cells and molecules present within tumours or other tissues.^12^, ^13^ In the context of the tumour microenvironment (TME), pivotal roles are played by immune cells, including T cells and natural killer cells.^14^ T cells, a cornerstone of the immune system, are broadly classified into CD4+ and CD8+ T cells^15^ and serve as key effectors in recognizing and eradicating pathogens, along with regulating aberrant cell growth.^16^ The immune microenvironment orchestrates T‐cell activity through diverse mechanisms. Regulatory T cells (Tregs), as suppressor immune cells within TME, restrain the activity of other immune cells, including dampening T‐cell responses.^17^ Concurrently, immunosuppressive entities within the TME may impede the normal functioning of T cells.^18^ Hence, an investigation into the signature genes linked with infiltrating T cells (ITSGs) in RCCC presents a potential avenue for elucidating innovative prognostic methodologies and deciphering the immune milieu within tumour cohorts.

Single‐cell RNA sequencing stands as a high‐throughput analytical methodology dedicated to scrutinizing gene expression within individual cells.^19^, ^20^ Tumour tissues encompass a diverse array of cell populations, including tumour cells, immune cells and vascular cells, among others.^21^ The application of single‐cell RNA sequencing facilitates the elucidation of gene expression patterns across distinct cell subpopulations within a tumour, thereby fostering a more thorough comprehension of tumour heterogeneity.^22^, ^23^

The emergence of spatial transcriptomics marks a pivotal transition in our understanding of the heterogeneous landscape of kidney cancer. Departing from traditional transcriptomic techniques, spatial transcriptomics not only interrogates the gene expression profiles of individual cells but also unveils the intricate spatial relationships and interactions among heterogeneous cell populations.^24^, ^25^ Harnessing this innovative methodology has revealed nuanced functional dynamics and intercellular dialogues within renal cancer tissues, thereby enriching our understanding of the origins of heterogeneity in this malignancy. These insights hold profound implications for the development of targeted therapeutic strategies, particularly those tailored to address the complexities associated with rare or specialized cell subpopulations.

In our pursuit, we have combined the capabilities of single‐cell sequencing with machine learning methodologies to delve into the prognostic implications of infiltrating T cells in KIRC. Our objective encompasses not only unravelling their role in the immune microenvironment but also predicting responses to drug therapies. A distinctive facet of our approach involves the innovative construction of a prognostic model grounded in ITSGs. The overarching aim is to deploy this novel model for precise and personalized prognostic predictions, thereby furnishing a scientific underpinning for the enhancement of treatment and management strategies for KIRC. By delving deeper into the intricate relationship between ITSGs and the evolution of KIRC, we aspire to chart new trajectories and generate innovative concepts for the future development of therapeutic interventions. This concerted effort seeks to contribute meaningful insights that transcend existing paradigms, offering a potential roadmap for advancing the landscape of therapeutic approaches in KIRC.

## 2 METHODS AND MATERIALS

### 2.1 Data sources for single‐cell data, spatial transcriptome data and bulk transcriptome data

**FIGURE 1:**
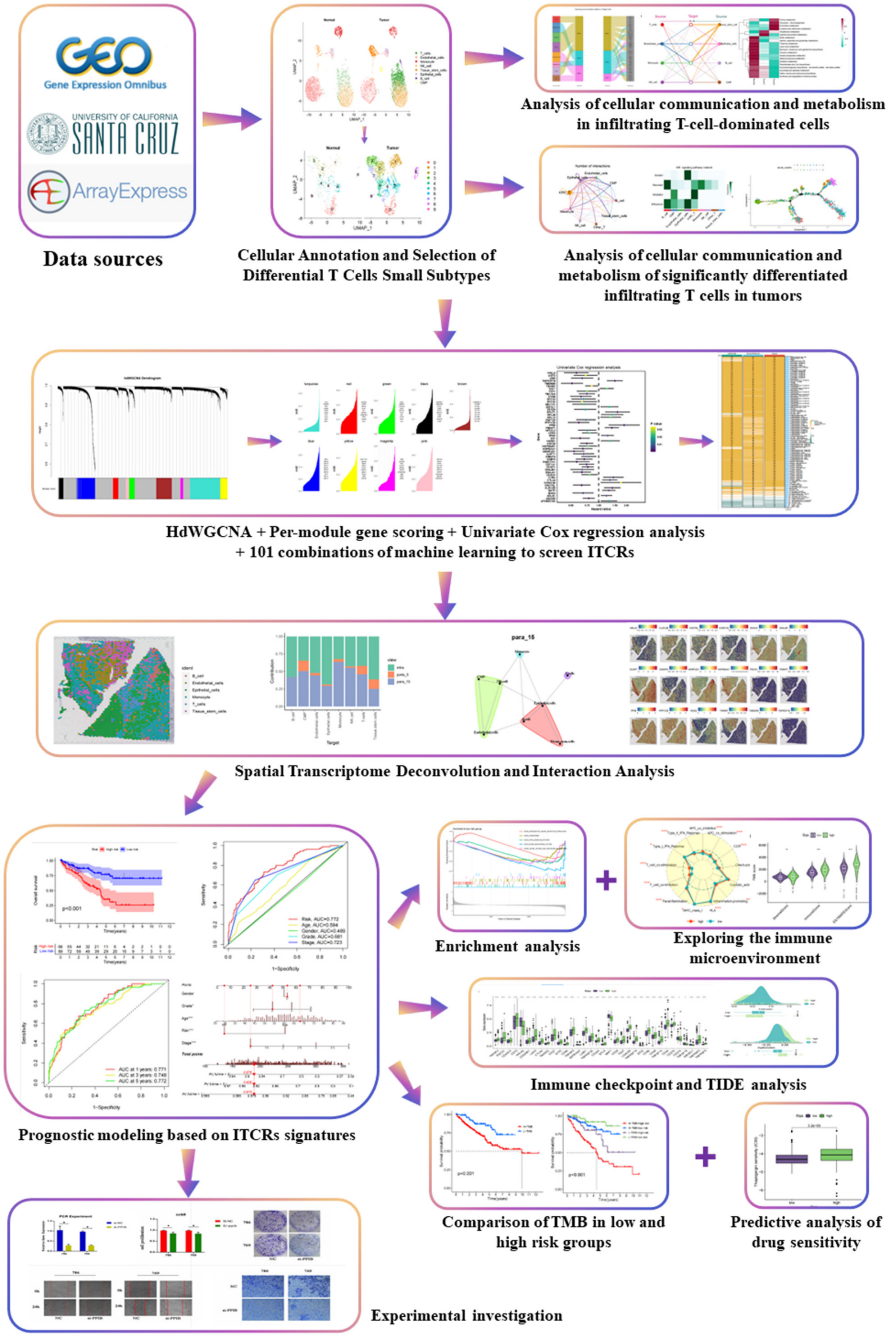
Flowchart demonstrating the research idea of this paper.

The methodological framework of this study is elucidated through a comprehensible flowchart, encapsulating our thought process (Figure 1). For single‐cell analysis, we procured a dataset of KIRC (GSE210038) from the Gene Expression Omnibus (GEO) database (https://www.ncbi.nlm.nih.gov/geo/). This dataset comprised two tumour tissue samples (GSM6415686 and GSM6415687) and one paracancerous tissue sample (GSM6415694). Spatial transcriptome data (GSM6415705 and GSM6415706) were obtained from GEO. Simultaneously, Gene expression profiles were extracted from 535 samples of KIRC and 72 normal samples, sourced from the Xena database (https://xenabrowser.net/hub/). To facilitate robust analysis, we judiciously divided the KIRC cohort into a training set and an internal test set, adhering to a ratio of 7:3. Additionally, we accessed 101 KIRC samples from the E‐MTAB‐1980 dataset of the Arrayexpress database (https://www.ebi.ac.uk/biostudies/arrayexpress/). Following data curation, seven samples were excluded due to missing data, leading to a final set of 94 KIRC samples designated for external validation. The collected clinical data encompassed information pertaining to patients' age, gender, STAGE, GRADE and pathologic TNM staging. For detailed data sources, please see Table S1.

### 2.2 Single‐cell dimensionality clustering and annotation

The single‐cell RNA sequencing data from KIRC underwent comprehensive analysis utilizing the R package ‘Seurat’.^26^ In the initial phase, we assessed the quality of cells, excluding those with either excessively high or low RNA feature counts and a heightened percentage of mitochondrial RNA. Subsequently, we identified highly variable genes through the ‘FindVariableFeatures’ function and conducted single‐cell RNA sequencing analysis employing the ‘harmony’ R package.^27^ The ‘FindVariableFeatures’ function was once again utilized for gene screening, while the ‘harmony’ R package facilitated the normalization of single‐cell data. The highly variable genes, upon normalization, underwent dimensionality reduction clustering analysis employing the Uniform Manifold Approximation and Projection method. The identification of cell types was conducted through the utilization of the ‘SingleR’ package^28^ for cluster annotation. This process was iterated to discern key subgroups, selecting cell groups exhibiting a significant differences to refine our understanding of the underlying cellular heterogeneity in KIRC.

### 2.3 Cell communication and proposed timing analysis

For a comprehensive exploration of signalling molecules between cells and a profound understanding of immune cell functionality in KIRC, we employed the ‘CellChat’ package within the R software environment.^29^ Concurrently, to delve into the heterogeneity of infiltrating T cells, we scrutinized the signalling pathways between infiltrating growth T cells and other T cells, immune cells and tissue cells utilizing the ‘cellchat’ R package. Simultaneously, the ‘monocle’ R package was utilized for the analysis of cellular developmental trajectories of T cells in a proposed time series.^30^ This integrated approach enabled us to unravel intricate cellular communication networks and delineate the temporal progression of T‐cell development within the context of KIRC.

### 2.4 HdWGCNA to obtain co‐expression modules

‘hdWGCNA’ R package is specifically designed for the integration and analysis of high‐dimensional single‐cell transcriptome data, aiming to discern patterns, associations and biological significance.^31^ The process involved the aggregation of neighbouring cells to form metacells, construction of sparse matrices for individual cells, the establishment of neighbouring matrices using TestSoftPowers to derive soft thresholds, transformation of these thresholds into Topological Overlap Matrix (TOM) matrices and generation of modules through hierarchical clustering tree‐based methodologies. The delineation of modules employed mirror tests, hierarchical clustering and dynamic tree‐cutting. Module definition was further refined using Module Affiliation Attributes and Genetic Significance to evaluate correlations between modules and clinical features. This approach facilitated the extraction of co‐expression modules, providing a structured framework to discern underlying biological relationships within the high‐dimensional single‐cell transcriptome data.

### 2.5 Screening for high‐performance ITSGs

ITSGs bearing prognostic significance were identified through a dual approach involving one‐way Cox regression and machine learning techniques. A comprehensive combinatorial analysis employing 10 distinct machine learning algorithms was conducted. The optimal algorithm, determined based on the highest C‐index value within each combination, was selected to screen for ITSGs for subsequent construction of prognostic models. We selected the best CoxBoost + Enet algorithm from different combinations of 10 machine learning algorithms based on the value of the C‐index. CoxBoost is a Cox proportional risk model based on Boosting technique, which improves the performance of the model by iteratively constructing multiple Cox models and combining their prediction results, whereas Elastic Net (Enet) is a regularization method that combines Lasso and Ridge regression to strike a balance between feature selection and predictive power. The risk score calculation for KIRC patients involved the use of the formula: $f\left(z\right)=\sum_{k=1}^{n}\text{Coef}\left(k\right)\times \text{Expr}\left(k\right)$. In this equation, *f*(*z*) represents the risk score, Coef(k) denotes the model coefficients, and Expr(k) signifies the expression level of ITSGs. The risk score calculation was implemented for KIRC patients, leading to the stratification of the bulk transcriptome dataset into groups characterized by high risk and low risk, determined by the median risk score. This stratification facilitated a nuanced understanding of the prognostic implications associated with the identified ITSGs in the context of KIRC.

### 2.6 Deconvolution analysis and spatial interactions between spatial transcriptome data and single‐cell data

Spatial deconvolution analysis, which integrates spatial transcriptome data with single‐cell sequencing data, stands as a pivotal technique facilitating the elucidation of cellular composition within heterogeneous samples. The amalgamation of cellular diversity from single‐cell sequencing and spatial information from transcriptome data is achieved through the utilization of the ‘spacexr’ R software package (https://github.com/dmcable/spacexr).^32^ Initially, this process entails the construction of a robust cell type decomposition (RCTD) object, wherein a reference of single‐cell transcriptome data augmented with annotation information is juxtaposed with the spatial transcriptome data encapsulated within a SpatialRNA object. Subsequently, leveraging the least squares method, estimates pertaining to gene expression patterns and cell type proportions within the spatial transcriptome data are derived. Furthermore, the outcomes of deconvolution analysis furnish valuable inputs for subsequent investigations utilizing the ‘mistyR’ R software package, enabling the exploration of spatial cellular interactions and the assessment of intercellular proximity within tissue contexts.

### 2.7 Construction and evaluation of prediction model

To enhance the clinical utility of prognostic predictions for KIRC, we leveraged the R package ‘rms’ in conjunction with ITSGs to construct a predictive model specific to KIRC. This model was visually presented in the form of a Nomogram. By amalgamating multiple predictive factors, including age, gender, clinical stage and risk score, this Nomogram provides comprehensive information, aiding in medical decision‐making and facilitating the formulation of individualized treatment plans. To assess the performance of the Nomogram, calibration curves and decision curves were employed for visualization and analysis. These metrics not only enhance the accuracy of clinical management but also contribute to a more precise prognostic assessment for KIRC patients. The Nomogram, through its integration of diverse predictive factors, serves as a valuable tool for clinicians, offering an insightful and holistic perspective for improved patient‐specific medical interventions and prognostic evaluations.

### 2.8 Enrichment analysis

To gain comprehensive insights into the expression levels of ITSGs across diverse functional pathways, we employed the R package ‘clusterProfiler’.^33^ Gene Set Enrichment Analysis (GSEA) of single genes was conducted to unveil their associations with specific pathways. Additionally, the ‘GSVA’ R package^34^ facilitated Gene Set Variation Analysis (GSVA) for a nuanced exploration of gene set variations. To delve deeper into the functional implications of ITSGs, we utilized ‘clusterProfiler’ for GO (Gene Ontology) Enrichment and KEGG (Kyoto Encyclopedia of Genes and Genomes) pathways. These analyses provided valuable insights into the pertinent gene functions and pathways associated with infiltrating T‐cell signatures, contributing to a more profound understanding of their roles in the context of KIRC.

### 2.9 Immune infiltration and TME analysis

The robust CIBERSORT algorithm, leveraging gene expression matrices, was harnessed as a potent tool for evaluating immune cell composition. Employing CIBERSORT, we quantified the relative proportions of 22 immune cells in KIRC patients and normal subjects. Furthermore, seven immunization algorithms, including XCELL, TIMER, QUANTISEQ, MCPCOUNTER, EPIC, CIBERSORT‐ABS and CIBERSORT, were deployed to ascertain the correlation between the risk score and the abundance of infiltrating immune cells. The TME scores for KIRC, encompassing IMMUNOLOGICAL scores, STOMAL scores and ESTIMATE scores, were computed using the ‘ESTIMATE’ R package. Differential analyses of immune checkpoints between patients in the high‐risk and low‐risk groups were conducted. Additionally, correlations between ITSGs and immune checkpoints were explored using the ‘limma’ package. To gauge tumour immune dysfunction and exclusion (TIDE) scores for KIRC, we utilized the online platform (http://tide.dfci.harvard.edu/). This multifaceted analysis aimed to unravel the intricate interplay between immune infiltration, the tumour microenvironment and the prognostic implications associated with ITSGs in KIRC.

### 2.10 Tumour mutations

A meticulous examination of the frequency and distribution of somatic mutations in KIRC was conducted using the ‘maftools’ R package.^35^ This package, renowned for its versatility, facilitated the visualization of tumour‐related mutation patterns, including coexistence and mutual exclusion. TCGA‐KIRC patients underwent stratification into four distinct groups, delineated by the median risk score of ITSGs and the median tumour mutation burden (TMB). A subsequent comparative analysis was carried out to discern survival differences among the groups. This analysis, focused on the median risk score and TMB values of each group, aimed to unravel the intricate interplay between ITSGs, tumour mutations and their collective impact on the prognosis of KIRC.

### 2.11 Drug sensitivity analysis

For the evaluation of immunotherapy response and the effectiveness assessment of frequently utilized chemotherapeutic drugs in KIRC patients, the ‘pRRophetic’ R package was utilized.^36^ This package facilitated the prediction of changes in drug sensitivity among KIRC patients, particularly concerning commonly utilized chemotherapeutic agents, based on the ITSGs. The predictive model is contingent upon the half‐maximal inhibitory concentration (IC50) derived from KIRC patients. This approach serves as a valuable tool for elucidating potential therapeutic responses and tailoring treatment strategies in the context of KIRC.

### 2.12 Cell cultures and transient transfections

Cultivation of human kidney clear cell carcinoma cell lines, specifically 786‐O and Caki‐2, alongside the human renal proximal tubular epithelial cell line HK‐2, was performed in Dulbecco's Modified Eagle's Medium (DMEM, GIBCO). The culture medium received supplementation with 10% fetal bovine serum (FBS; Hyclone), alongside 100 U/L penicillin and 100 mg/L streptomycin (Thermo Fisher). Cell cultures were maintained at 37°C with a 5% CO_2_ atmosphere. In the context of transient transfection, both negative control (NC) and BRD9 siRNA (RiboBio, Guangzhou, China) were transfected into colorectal cancer (CRC) cells using Lipofectamine 3000 (Invitrogen, Carlsbad, CA, USA), following the manufacturer's instructions. This experimental setup laid the foundation for investigating the impact of BRD9 knockdown on CRC cells and allowed for the exploration of potential therapeutic targets in KIRC.

### 2.13 PCR experiment

Total RNA extraction was performed employing the RNA Easy Fast Tissue/Cell Kit (Sichuan Jielaimei Technology Co., Ltd), following the manufacturer's protocol. Real‐time PCR reactions were conducted using SuperReal PreMix Plus reagents (Sichuan Jielaimei Technology Co., Ltd) in conjunction with the StepOnePlus Real‐Time PCR System. The real‐time PCR reaction protocol comprised an initial pre‐denaturation phase at 95°C for 15 min, succeeded by 40 cycles involving denaturation at 95°C for 10 s, annealing at 72°C for 20 s and extension at 60°C for 20 s.

### 2.14 CCK‐8 assay

Evaluation of cell viability was conducted using the Cell Counting Kit‐8 (CCK‐8) assay. Initially, cells were seeded at a density of 1500 cells per well in 96‐well plates, each containing 200 μL of complete medium, and were incubated at 37°C. Upon completion of each experimental condition, 20 μL of CCK‐8 reagent (Beyotime, Shanghai, China) was added to individual wells. Subsequent to the incubation of the plates for an additional 2 h, the determination of optical density values (OD_450 nm_) was executed utilizing a microplate reader.

### 2.15 Plate cloning assay

Within the context of plate cloning experiments, the initial steps involve retrieving target cells from a culture vessel to assess their physiological resilience. Subsequent to this, the cells are dispersed into individual entities through a gentle centrifugation process, ensuring that each clone originates from a single‐cell source. Facilitating the cloning process necessitates the prior application of a growth medium, replete with essential constituents, onto a pre‐coated petri dish or culture plate, thereby establishing an environment conducive to cellular proliferation. Following this preparatory step, the dispersed cells are introduced onto the pre‐prepared medium at a density deemed appropriate for the intended purpose. Subsequently, the petri dishes or plates are transferred into a cell culture incubator, where they undergo cultivation under conditions meticulously maintained to uphold optimal temperature and controlled CO2 levels.

### 2.16 Wound‐healing assay

In order to assess the migratory behaviour of KIRC cells, we conducted a wound‐healing assay. KIRC cells, subjected to transfection and cultivation in 6‐well plates, were maintained at 37°C until reaching an approximate confluency of 80%. Subsequently, precise wounds were generated within the cellular monolayer utilizing a sterile 200 μL pipette tip. Upon induction of the wounds, the cells underwent dual rinses with phosphate‐buffered saline to eliminate any residual debris. Following this, the culture medium was replaced with serum‐free medium to mitigate potential confounding variables. The migratory dynamics of cells infiltrating into the wounded area were meticulously monitored at both 0 and 24‐h intervals utilizing an Olympus inverted microscope.

### 2.17 Transwell assay

In the Transwell assay, a uniform distribution of 1 × 10^5^ cells occurred across two distinct cell chambers: a Matrigel‐coated chamber (BD Biosciences, San Jose, CA) designated for invasion assays and an uncoated chamber designated for migration assays. The upper chamber was filled with medium devoid of serum, whereas the lower chamber was loaded with complete DMEM medium. After a 24‐h incubation period, cells that exhibited successful migration or invasion through the membrane were fixed using 4% paraformaldehyde and subsequently subjected to staining with 0.1% crystal violet. Cell number quantification was conducted through light microscopy (Thermo Fisher, Waltham, MA, USA).

### 2.18 Statistical analysis

Our extensive statistical analysis and data processing were conducted using R version 4.1.3 and Strawberry Perl version 5.30.0. To gauge the statistical significance of survival analyses, we employed Kaplan–Meier curves and conducted log‐rank tests. For variables adhering to a normal distribution, differences were quantified using *t*‐tests or one‐way ANOVA, as appropriate. In instances where data did not conform to a normal distribution, the Wilcoxon test or Kruskal–Wallis test was utilized to assess differences. Analysis of data derived from the CCK‐8 assay was conducted utilizing GraphPad Prism software (version 8.3.0). The results were expressed as the mean ± standard deviation (SD) based on three independent experiments and subjected to analysis via ANOVA. A rigorous significance threshold of <0.05 was upheld for all statistical analyses.

## 3 RESULTS

### 3.1 Acquisition of key subpopulations of infiltrating T cells from KIRC single‐cell dataset

**FIGURE 2:**
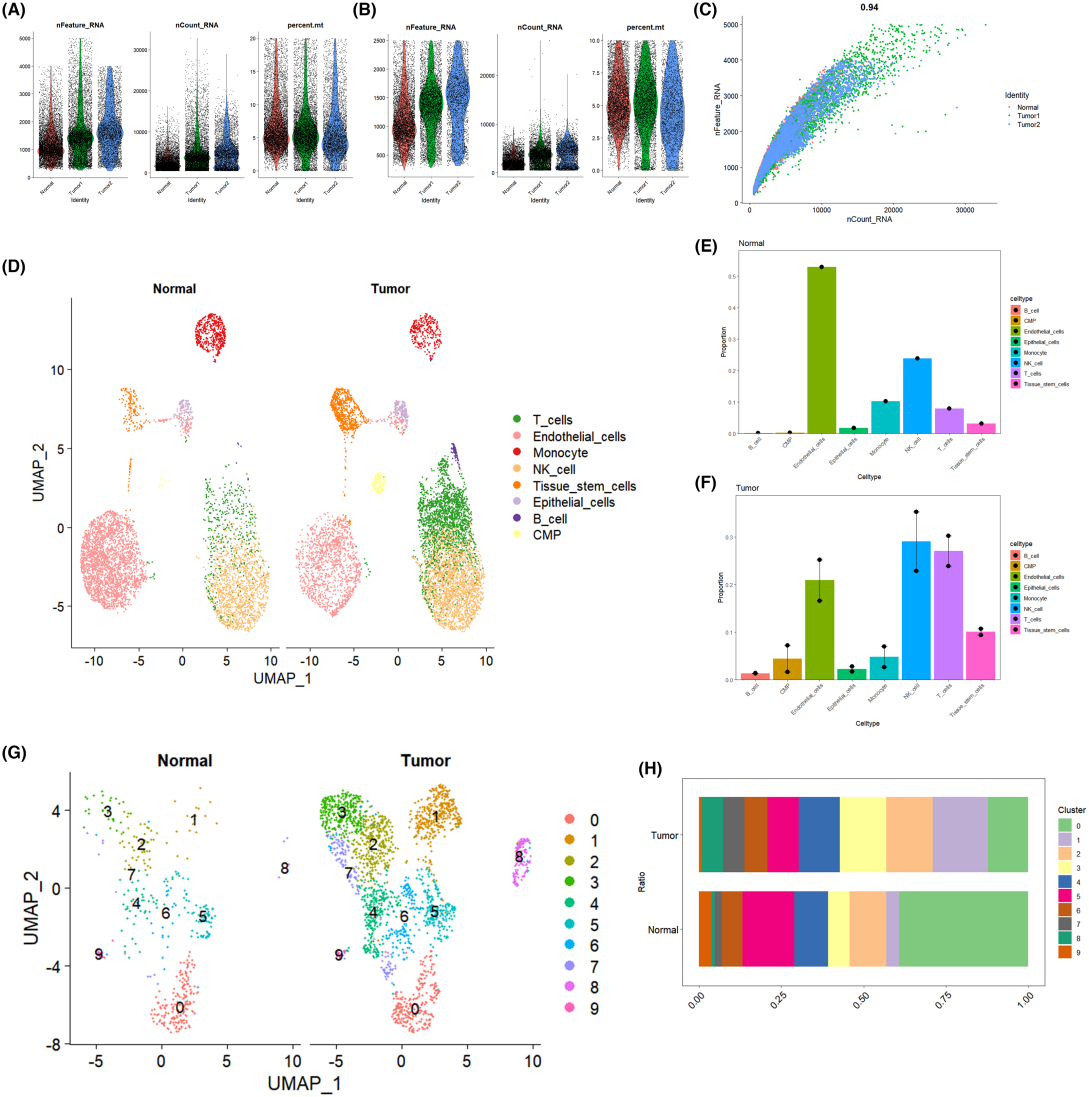
Cell annotation and acquisition of key small subpopulations of infiltrating T cells. (A, B) Shows the comparison of single‐cell data processing before and after quality control. (C) Correlation coefficient between ‘nFeature_RNA’ and ‘nCount_RNA’ after quality control. (D) UMAP plot showing the distribution of various cell types in tumour vs. normal after annotation. (E, F) Bar graphs showing the percentage of each cell type in normal and tumour. (G) UMAP plot showing the distribution of small subpopulations of T cells in normal and tumour after dimensionality reduction clustering. (H) Bar graph showing the percentage of each small subpopulation of T cells in tumour and normal.

The KIRC single‐cell dataset underwent preprocessing using the ‘Seurat’ R software package to illustrate details such as ‘nFeature_RNA’, ‘nCount_RNA’ and ‘percent.mt’ in the single‐cell dataset before quality control (QC) (Figure 2A). Quality control measures were implemented to filter out cells expressing more than 2500 or fewer than 200 genes, as well as cells with a mitochondrial gene content ratio exceeding 10%. This ensured the retention of cells with 200 < nFeature_RNA < 2500 and percent.mt < 10 (Figure 2B). A satisfactory correlation coefficient of 0.94 between ‘nFeature_RNA’ and ‘nCount_RNA’ post‐quality control indicated the overall favourable cell quality (Figure 2C). Following normalization, identification of highly variable genes and matrix expression normalization, the process of dimensionality reduction clustering commenced. The KIRC cell population was categorized into 14 clusters and annotated using the ‘celldx’ software package. Eight distinct cell types were identified, including T cells, endothelial cells, monocytes, NK cells, tissue stem cells, epithelial cells, B cells and CMPs. The distribution of these cells in the tumour group and normal group was visualized using UMAP (Figure 2D). The proportions of the eight cell types in the tumour and normal groups were plotted, revealing a notable increase in infiltrating T cells (Figure 2E,F). Specifically, T cells were individually extracted for dimensionality reduction clustering analysis, resulting in the classification of 10 smaller subgroups (Figure 2G). Bar scale graphs for comparison highlighted that subpopulations 1, 2, 3, 4, 7 and 8 exhibited the most significant growth in the tumour group, indicating their high research significance (Figure 2H).

### 3.2 CellChat deciphering T cell‐focused cellular communication in the tumour microenvironment of KIRC

**FIGURE 3:**
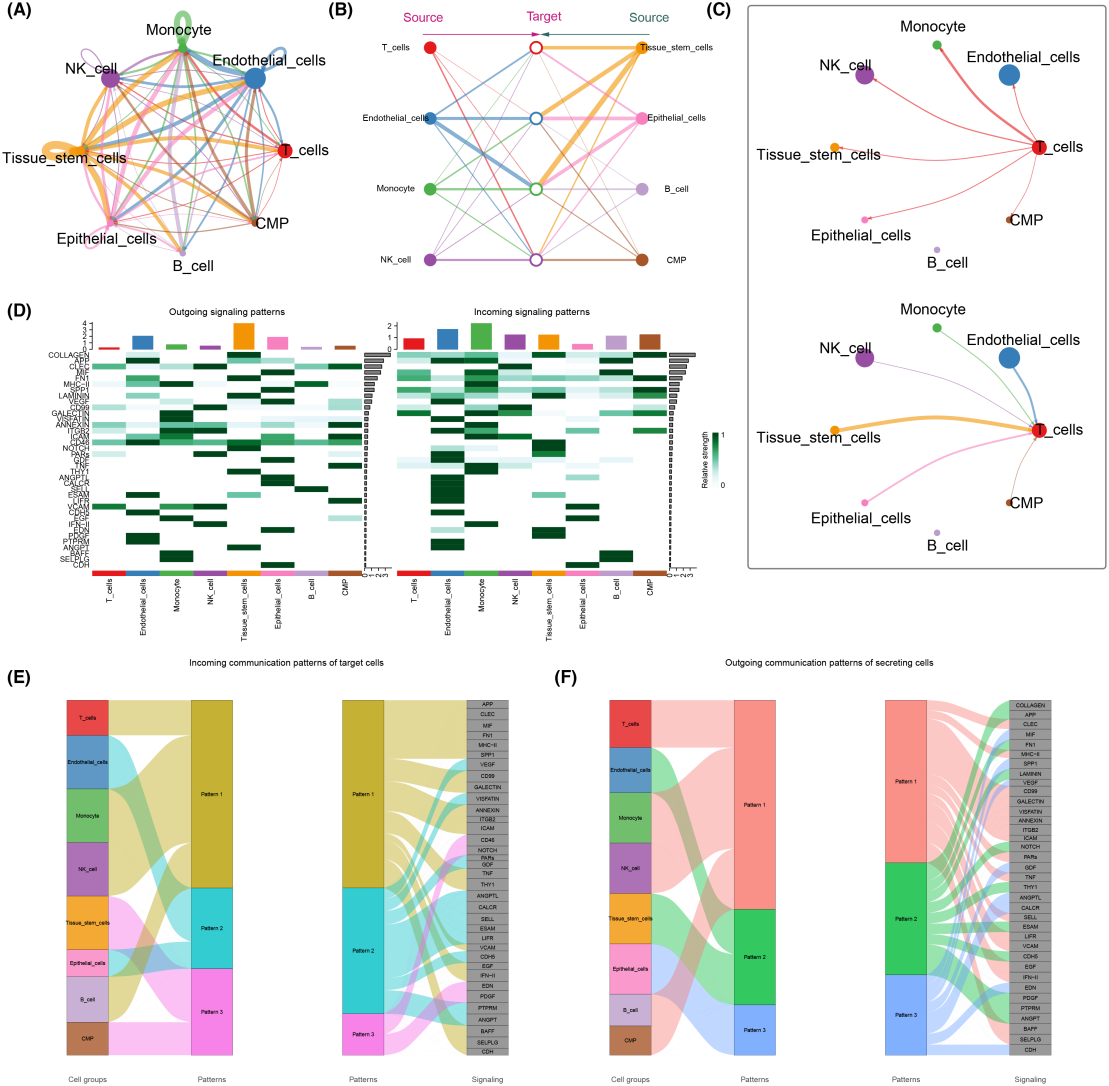
Communication among cell subpopulations. (A) Number of interactions among cell subtypes. (B) Hierarchy and structure among cell subtypes. (C) String diagram of T‐cell interactions with other cells. (D) Heat map showing the strength of efferent and afferent signalling pathways in different cell subpopulations. (E, F) River diagrams showing the relative positions and connectivity of different cells in the receptive and efferent signalling networks.

To gain deeper insights into the role of T cells in the tumour microenvironment (TME) of KIRC, we utilized the CellChat software package for the prediction and analysis of T cell‐focused intercellular communication networks. Annotated tumour tissue cells were initially extracted from Seurat objects and subsequently imported into the human ligand‐receptor database within the software package. CellChat employs a simulation approach to model the probability of intercellular communication by integrating gene expression data with known interactions between signalling ligands, receptors and cofactors. The model utilizes various modes of action to derive the number of interactions between different cell types, considering the number of receptors ligands and the strength of communication (Figure 3A). The hierarchy and structure of the relationship between immune cells and tissue cells were analysed (Figure 3B), and the frequency of T cells receiving and sending signals was illustrated (Figure 3C). In the OUTGOING signalling patterns, we observed that C‐type lectin domain family (CLEC), CD99, ANNEXIN, CD46 and vascular cell adhesion molecule (VCAM) exhibited strong correlations with T cells. Conversely, the INCOMING signalling patterns revealed strong correlations with T cells for COLLAGEN, macrophage migration inhibitory factor (MIF), fibronectin 1 (FN1), secreted phosphoprotein 1 (SPP1) and GALECTIN (Figure 3D). River diagrams depicted the relative positions and connectivity of different cells in the signalling network (Figure 3E,F). Notably, T cells, monocytes, NK cells and B cells functioned as a common Incoming communication pattern in signalling pathways such as amyloid beta precursor protein (APP), CLEC, MIF, FN1, major histocompatibility complex class I (MHC‐1I) and SPP1. Additionally, T cells, monocytes, NK cells and CMP acted as a common outgoing communication pattern in signalling pathways such as CLEC, CD9, GALECTIN, VISFATIN, ANNEXIN, integrin beta‐2 (ITGB2) and intercellular adhesion molecule (ICAM).

### 3.3 Mimetic time‐series analysis and cellular metabolic enrichment analysis at the single‐cell level of KIRC

**FIGURE 4:**
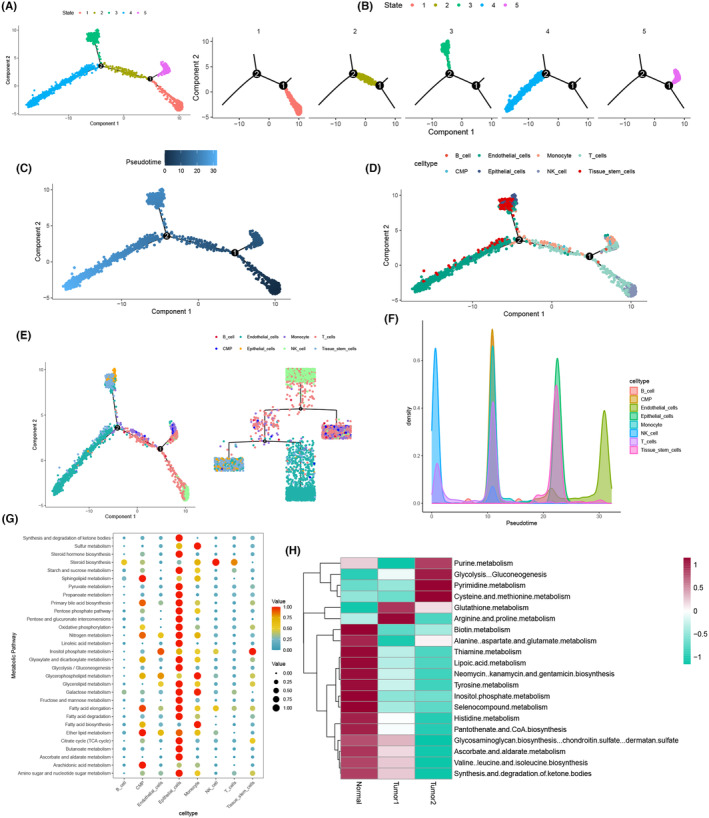
Proposed temporal analysis and cellular metabolism. (A, B) Proposed chronological analysis of individual states. (C–E) Developmental time and cell trajectories of each cell subpopulation of kidney renal clear cell carcinoma (KIRC). (F) Cell density of various cell subpopulations versus developmental time. (G) Metabolic analysis of different cells. (H) Metabolic analysis of T cells in three samples.

To infer the differentiation and evolutionary trends of KIRC tissue cells at the single‐cell level, we constructed intercellular trajectories, simulating the cellular development process through proposed time‐series analysis and cell trajectory analysis. The temporal distribution of tumour cells and their trajectory development were observed using the R package ‘monocle’, where each developmental stage was presented separately and divided into five states (Figure 4A,B), demonstrating the left‐to‐right proliferation pattern of KIRC at the single‐cell level (Figure 4C). Different cell types were represented by distinct colours, with T cells predominantly clustering on the rightmost side, exhibiting preferential growth compared with other cells (Figure 4D). The developmental trajectories of different cells were incrementally displayed, revealing a branching development of T cells during progression (Figure 4E). Different cell types exhibited varying cell densities across developmental stages, with T cells showing high‐density growth mainly in the anterior‐middle stag e (Figure 4F). Metabolic pathway enrichment analysis for different cells in KIRC highlighted pathways such as steroid biosynthesis, starch and sucrose metabolism, galactose metabolism, fatty acid elongation, and amino sugar and nucleotide sugar metabolism, demonstrating high association with T cells (Figure 4G). Additionally, metabolic analysis of T cells in three samples revealed varying degrees of metabolic enhancement in tumour samples compared with normal samples. Notably, pathways such as purine metabolism, pyrimidine metabolism, cysteine and methionine metabolism, glutathione metabolism, and arginine and proline metabolism displayed increased metabolic activity in tumour samples. Conversely, pathways including biotin metabolism, alanine aspartate and glutamate metabolism, thiamine metabolism, lipoic acid metabolism, neomycin kanamycin and gentamicin biosynthesis, tyrosine metabolism, inositol phosphate metabolism, selenocompound metabolism, histidine metabolism, and pantothenate and CoA biosynthesis exhibited reduced metabolic activity in tumour samples (Figure 4H).

### 3.4 Cell communication and proposed time‐series analysis of key small subpopulations of infiltrating T cells

**FIGURE 5:**
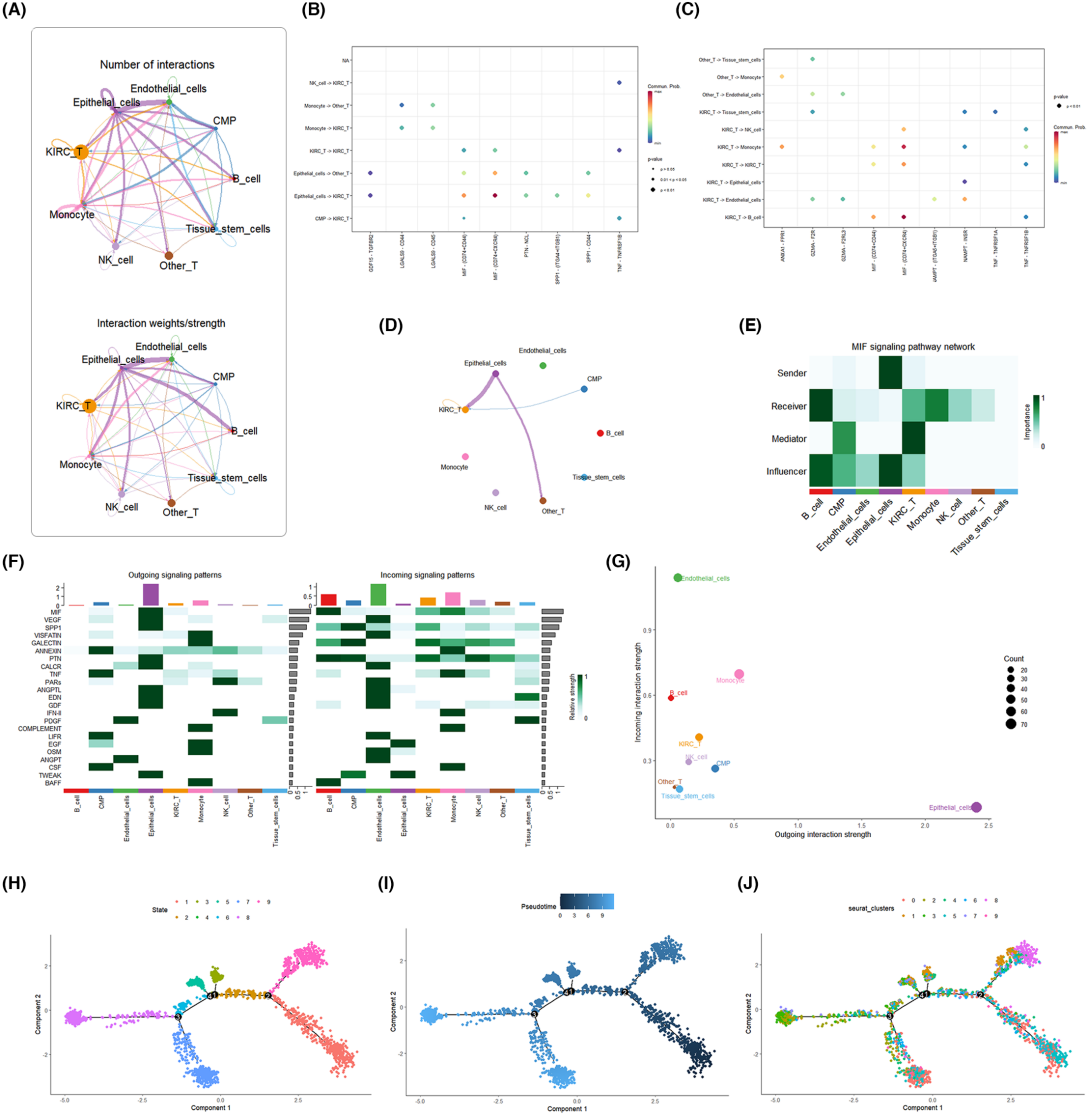
Cellular communication and proposed time‐series analysis of key small subpopulations of infiltrating T cells. (A) Number and strength of interactions between key small subpopulations of infiltrating T cells and other cells in the tumour microenvironment (TME). (B, C) Dot plots of communication pathways between key small subpopulations of infiltrating T cells and other cells. (D) String diagram of the migration inhibitory factor (MIF) pathway network. (E) Heat map of the MIF signalling pathway network. (F) Heatmap of the receptive and output signalling pathways between different cells after the addition of key small subpopulations of infiltrating T cells. (G) Scatter plot showing the distribution of different cell populations in the strength of efferent and afferent signalling interactions. (H‐J) Developmental time and cellular trajectories of small subpopulations ofT cells.

To delve deeper into the key small subpopulations of infiltrating growing T cells at the single‐cell level, we conducted cell communication analysis and proposed time‐series analysis centred around these subpopulations. The six most differentiated key subpopulations of infiltrating T cells in the KIRC tumour group (1, 2, 3, 4, 7 and 8) were designated as KIRC_T cells, while other types of T‐cell subpopulations were labelled as Other_T cells. Utilizing the ‘CellChat’ package, we analysed the number and strength of interactions between KIRC_T cells and other cell types (Figure 5A), identifying overexpressed genes and projecting ligands and receptors to the PPI network. Comparison of KIRC_T cells with Other_T cells in sending and receiving signals revealed a significant difference in the MIF signalling pathway, suggesting its crucial role in T‐cell communication (Figure 5B,C). MIF is known for its involvement in immune response and inflammation regulation, and its impact on the TME may influence tumour immune escape and progression. Chordal graphs illustrated the role of the MIF signalling pathway in cellular communication within tumour tissues, with the main targets being epithelial cells, CMP and Other_T cells (Figure 5D). In the MIF signalling pathway network, KIRC_T cells acted as receivers, mediators and influencers, with a notable mediator role in interactions with Other_T cells (Figure 5E). MIF appears to mediate interactions between KIRC_T cells and other cells in the TME, fostering complex interactions among T cells and other cell populations within the TME (Figure 5F). Scatter plots demonstrated the correlation between outgoing and incoming interaction strengths of KIRC_T cells with other cell subpopulations, reaffirming the role of KIRC_T cells as Mediators in signalling other cell subpopulations (Figure 5G). To infer the differentiation and evolutionary trends of T cells at the single‐cell level, trajectories were constructed among different T‐cell subpopulations to replicate the developmental process of T cells. The developmental trajectories of T cells were categorized into a total of nine states, with the overall T‐cell developmental time progressing from right to left (Figure 5H,I). Key subpopulations 1, 2 and 8 were mainly expressed in the mid‐developmental stage, while 3, 4 and 7 were primarily expressed in the end‐developmental stage (Figure 5J).

### 3.5 Screening of core genes of key T‐cell subpopulations using hdWGCNA

**FIGURE 6:**
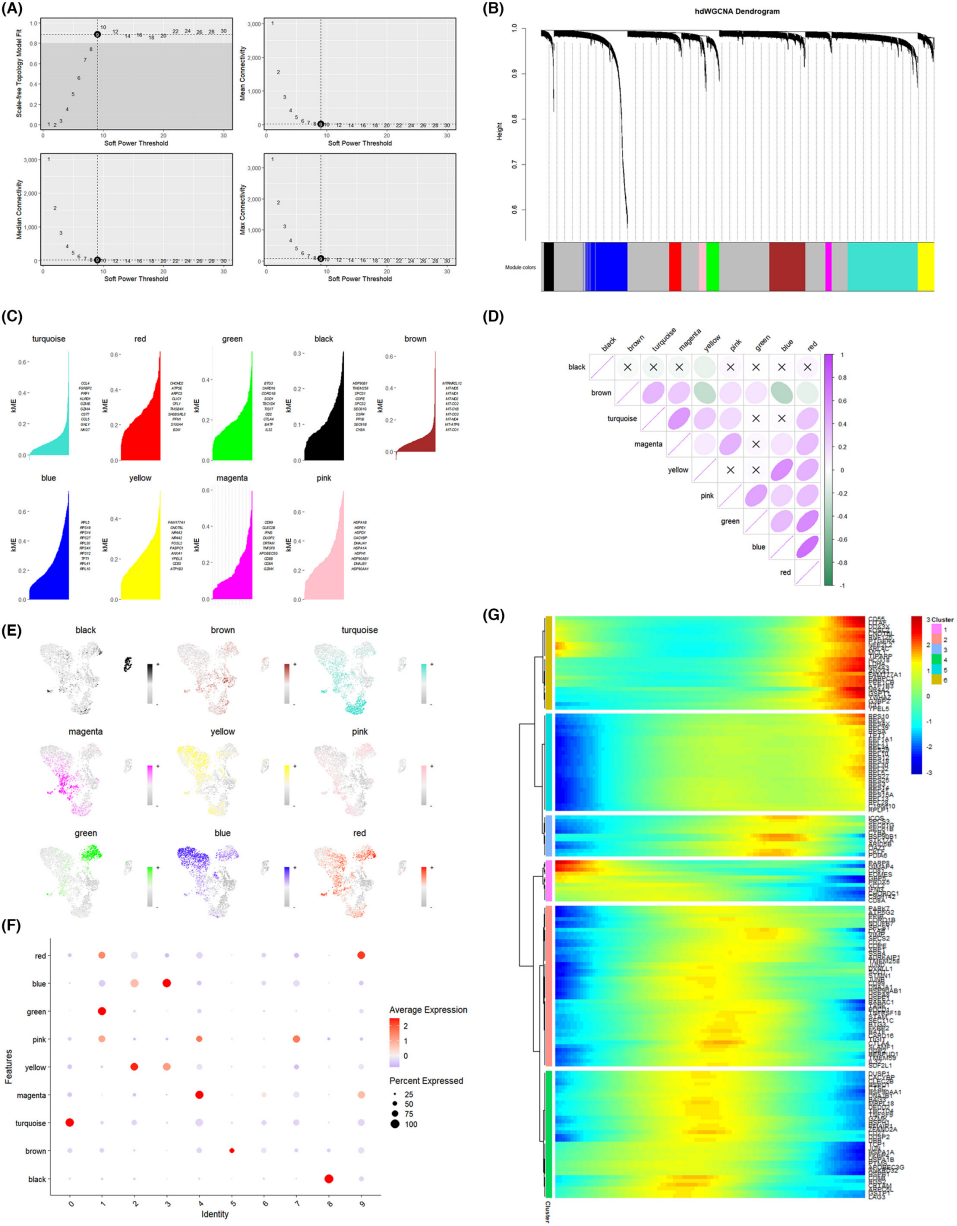
Screening of T‐cell key subpopulation core genes using high‐density weighted gene co‐expression network analysis (hdWGCNA). (A) Determination of the optimal soft threshold for performing clustering. (B) Clustering module diagram of hdWGCNA. (C) Scoring of genes in different modules. (D) Correlation plots between different modules. (E, F) Distribution of scored genes of different modules in T cells and small subpopulations of T cells. (G) Expression of core genes of key T‐cell subpopulations during the period of T‐cell development.

To delve into the intrinsic properties of KIRC single‐cell RNA‐seq data, we employed high‐dimensional weighted correlation network analysis (hdWGCNA) to discern gene expression differences between KIRC tumour samples and normal samples. This aimed to identify the core genes associated with key subpopulations of T cells. After excluding apparent abnormalities, subcellular clustering was performed on the single‐cell data, and the soft threshold of 9 was calculated using ‘TestSoftPowers’ to construct the co‐expression network (Figure 6A). The analysis produced nine colour modules along with the grey module (Figure 6B). The ‘Ucell’ package was utilized to score genes in each colour module, and the top 10 genes with the highest scores in each module were visualized (Figure 6C). The heat map depicted the correlation between modules (Figure 6D). The set of genes clustered in each module was mapped to the UMAP colour space, revealing the distribution of each gene module (Figure 6E). Scatter plots highlighted the colour modules within which each T‐cell subpopulation gene predominantly clustered. Specifically, T‐cell key subpopulations 1, 2, 3, 4, 7 and 8 corresponded to the modules ‘green’, ‘yellow’, ‘blue’, ‘magenta’, ‘pink’ and ‘black’, respectively (Figure 6F). The top 25 genes with the highest scores in each of the six modules were selected as T‐cell key genes. Most of these key genes exhibited high expression in the middle and late stages of T cells, aligning with the results of the proposed time‐series analysis (Figure 6G).

### 3.6 Multiple machine learning combinations to screen ITSGs and construct prognostic models

**FIGURE 7:**
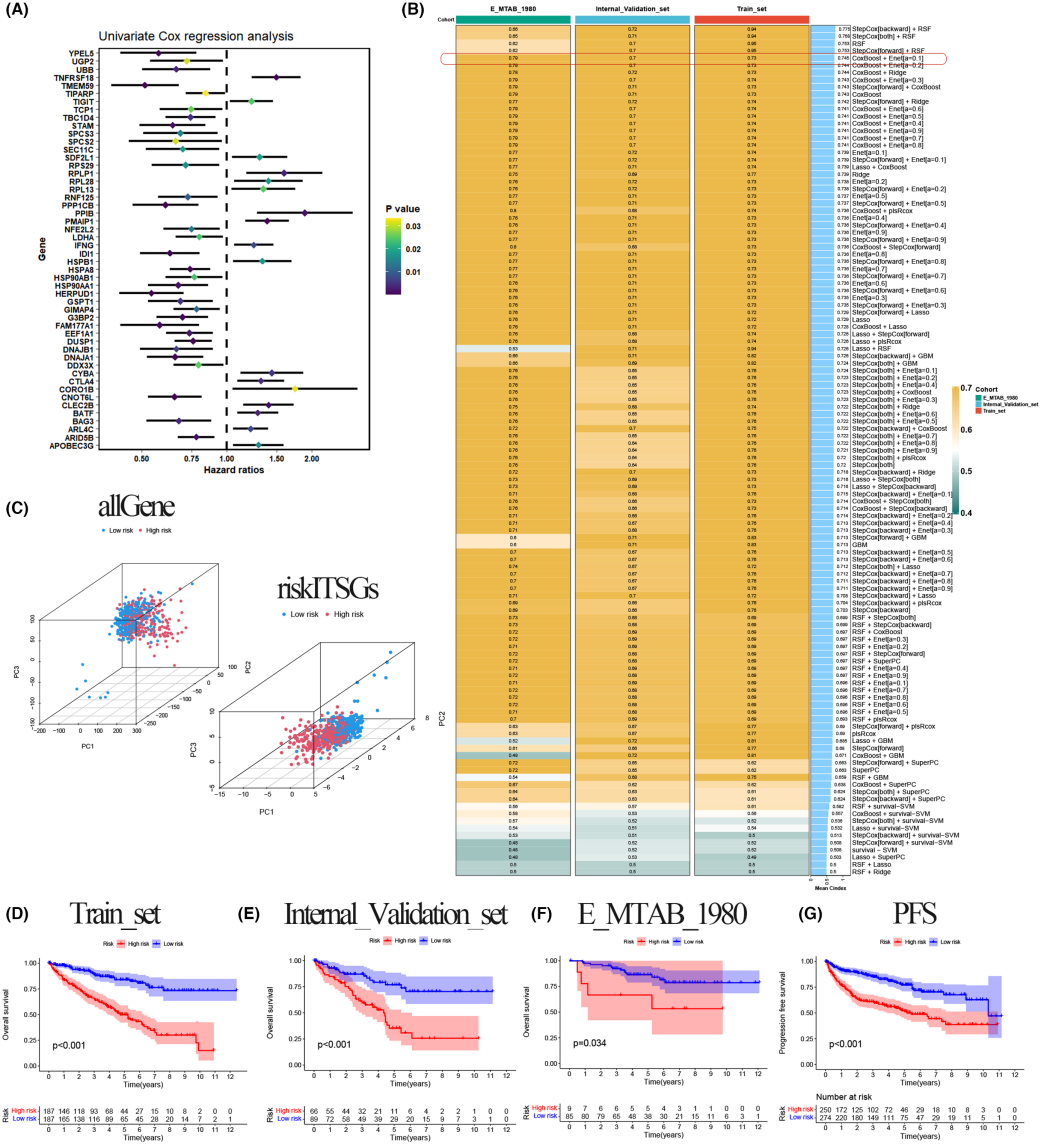
Screening of ITSGs and construction of prognostic models. (A) One‐way cox prognostic analysis. (B) C‐index values derived from 118 combinations of 10 machine learning algorithms. (C) Principal component analysis (PCA) plot of all genes versus risk ITSGs. (D–F) Survival curves for training set, internal validation set and external validation set. (G) Survival curves for predicting progression‐free survival (PFS).

We conducted a one‐way Cox prognostic analysis on the 150 core genes of key T‐cell subpopulations, revealing 53 genes with significant prognostic value for KIRC (Figure 7A). Subsequently, the TCGA data samples were randomly divided into a training set and an internal validation set (7:3 ratio), while the E‐MTAB‐1980 dataset from the Arrayexpress database served as the external validation set. The CoxBoost + Enet algorithm was selected as the best among 118 combinations from 10 machine learning algorithms based on C‐index values. Ultimately, 20 genes were identified to construct the prognostic model (Figure 7B). These 20 ITSGs included ARL4C, CLEC2B, CNOT6L, CORO1B, DDX3X, DNAJB1, DUSP1, GIMAP4, HERPUD1, HSP90AA1, PDCD1, PMAIP1, PPIB, PPP1CB, RGS2, RPL13, RPS29, TIPARP, TMEM59 and TNFRSF18. The expression of the selected 20 ITSGs was multiplied by the corresponding regression coefficients, and the results were summed to obtain the risk score for each sample. Based on the median value of the risk scores in the training set, the training set, internal validation set and external validation set were categorized into high‐risk and low‐risk groups. Principal component analysis of the risk model showed that ITSGs effectively categorized TCGA into high‐risk and low‐risk groups compared with all genes, forming two relatively independent clusters (Figure 7C). To assess the value of the constructed risk‐scoring model for KIRC patients in prognosis, survival curves were plotted for the training set, internal validation set and external validation set. The results demonstrated significant differences in overall survival between high‐ and low‐risk groups in the training set (Figure 7D, *p* < 0.001), internal validation set (Figure 7E, *p* < 0.001) and external validation set (Figure 7F, *p* = 0.034). The overall survival of KIRC patients in the low‐risk group was higher than that in the high‐risk group. Furthermore, progression‐free survival prediction for tumour samples in the overall TCGA revealed a significant difference between high‐ and low‐risk groups (Figure 7G, *p* < 0.001), with a higher survival rate observed in low‐risk patients.

### 3.7 Spatial transcriptome sequencing combined with ITSGs, RCTD deconvolution analysis and spatial interactions analysis

**FIGURE 8:**
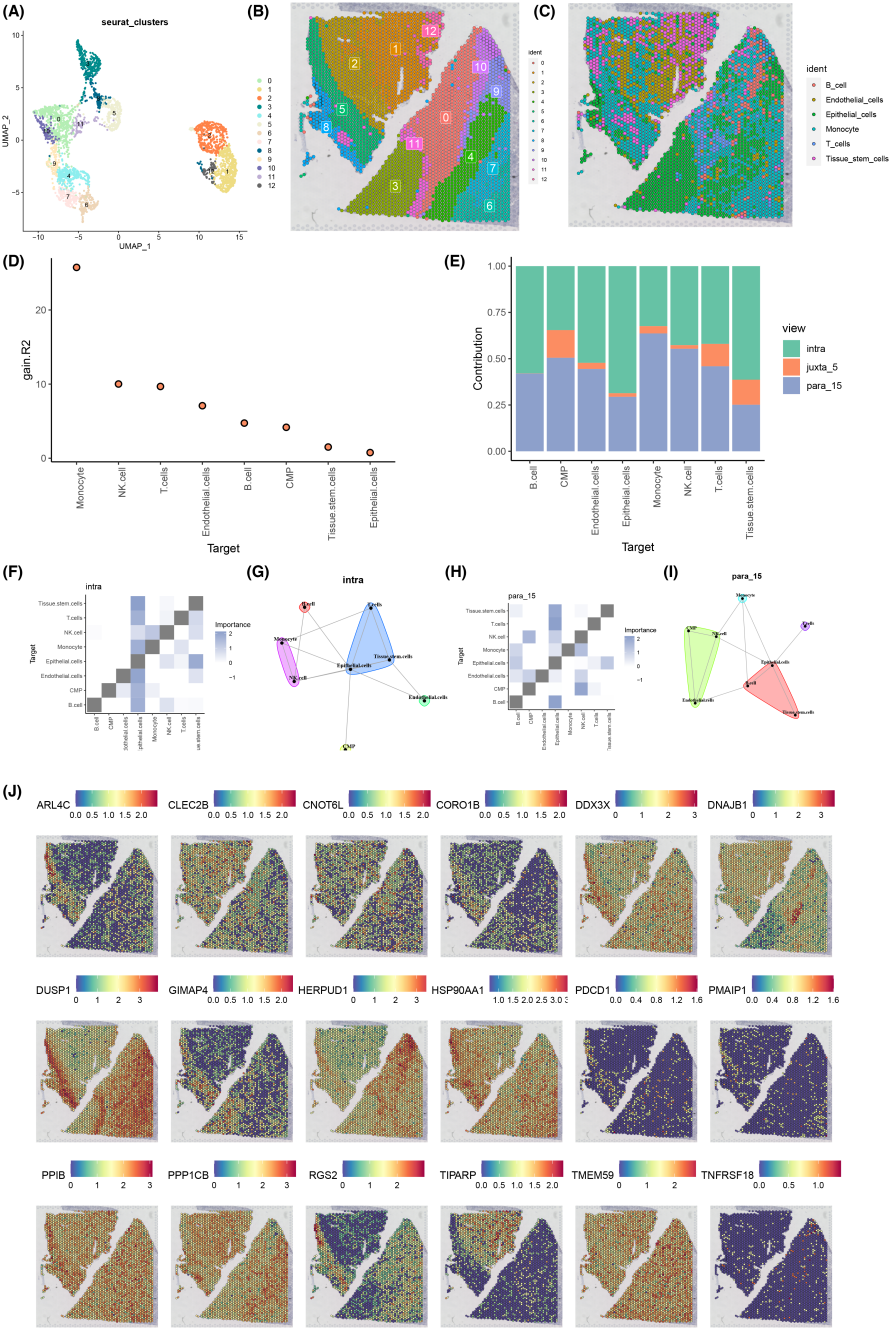
Spatial transcriptome deconvolution and interaction analysis. (A, B) Utilizing robust cell type decomposition (RCTD), the spatial transcriptome data of kidney renal clear cell carcinoma (KIRC) was stratified into 13 distinct cell populations. (C) RCTD deconvolution facilitated the mapping of cell types from single‐cell data onto spatial transcriptome slices, elucidating cellular heterogeneity within the tissue microenvironment. (D) Enhanced statistical analyses and correlation assessments across diverse cell types were achieved, providing insights into their functional dynamics. (E) Distribution histogram depicting the contributions of custom functions, namely intra, juxta_5 and para_15, towards determining cell importance metrics. (F) Heatmap representation delineating correlations among various cell types computed through intrafunction. (G) Analysis of spatial neighbourhood correlation between different cell types, calculated via intrafunction. (H) Heat map illustrating correlation patterns between distinct cell types derived from para_15 analysis. (I) Assessment of spatial neighbourhood correlation among different cell types, determined utilizing para_15 analysis. (J) Spatial distribution profile of ITSGs across spatial transcriptome tissue sections.

To investigate the spatial heterogeneity within tumours, we integrated spatial transcriptome data with single‐cell information for comprehensive analysis. Initially, we conducted dimensionality reduction clustering on the spatial transcriptome data, partitioning it into 13 distinct subgroups via UMAP analysis across tissue sections (Figure 8A,B). Subsequently, employing the RCTD, we delineated the cellular composition onto the spatial transcriptome slices, revealing six discernible cell clusters (Figure 8C). Leveraging the ‘mistyR’ R software suite, we executed spatial cell–cell interaction analyses, enhancing the statistical inference across various cell types (Figure 8D). Furthermore, employing the functionalities within the ‘mistyR’ R package, namely intra, juxta_5 and para_15, we scrutinized the contributions, interrelations and correlations among individual cell types. The contribution bar graphs effectively portray the magnitude of associations and interdependencies among diverse cell populations (Figure 8E). Notably, the proximity correlation heatmap, alongside spatial proximity correlations computed through the intra function, unveiled a robust correlation between T cells and epithelial cells, with monocytes exhibiting a significant correlation with tissue stem cells (Figure 8F,G). Conversely, distant correlation heatmaps and spatial proximity correlations derived from para_15 analysis demonstrated a pronounced correlation solely between T cells and epithelial cells (Figure 8H,I). Moreover, we scrutinized the expression profiles of ITSGs on spatial transcriptome tissue sections, revealing a strong expression of PPIB, PPP1CB, TMEM59 and HSP90A11 (Figure 8J).

### 3.8 Comparative study between risk scores and clinical features based on ITSGs and construction of nomogram for independent prognostic analysis

**FIGURE 9:**
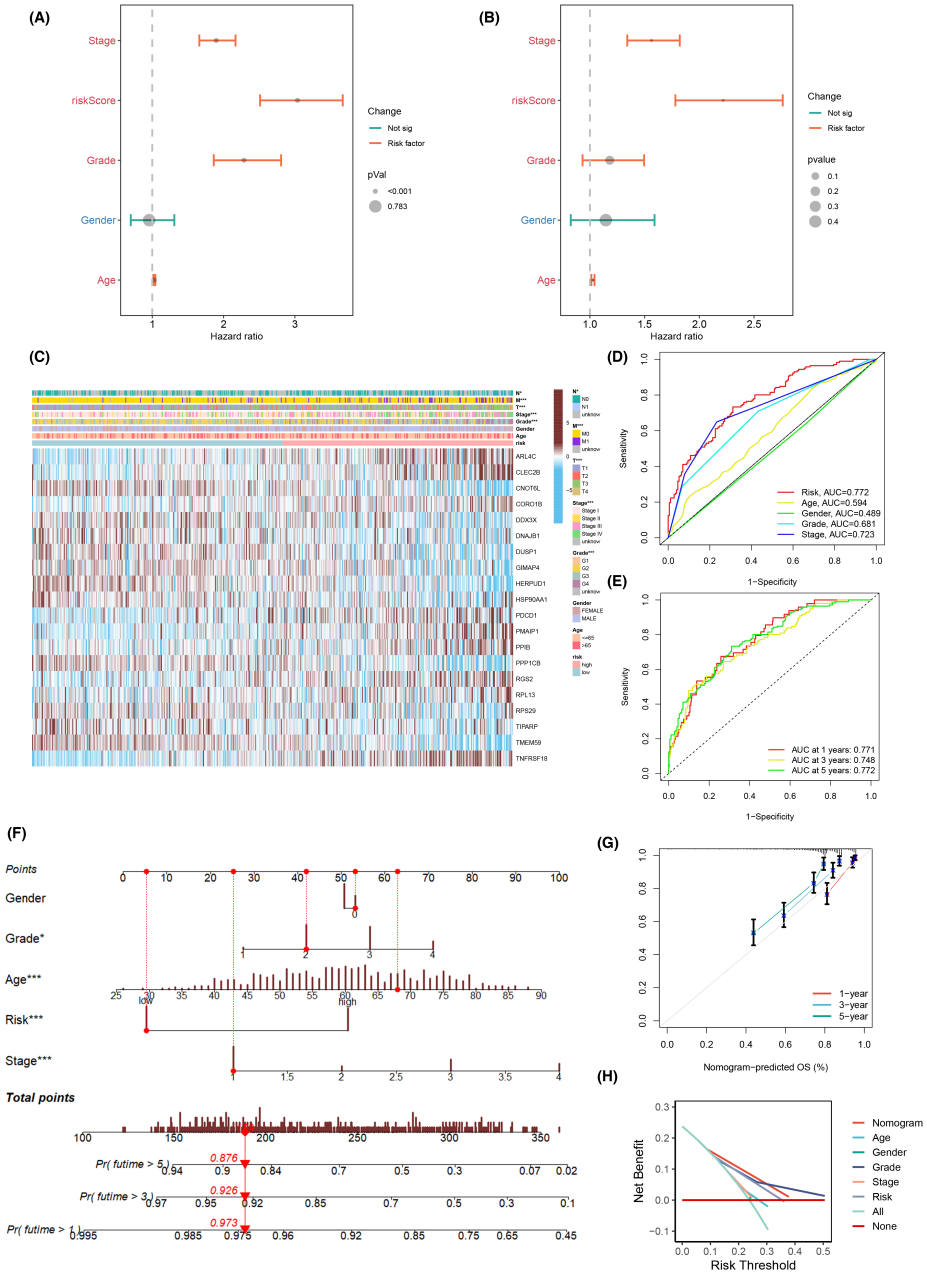
Analysis of independent prognostic factors and construction of nomogram. (A, B) Univariate independent prognostic analysis versus multivariate independent prognostic analysis. (C) Heat map demonstrating the association between risk scores and clinicopathologic features. (D) Receiver operating characteristic curve (ROC) curves of risk groupings versus each clinical characteristic. (E) ROC curves predicting 1‐, 3‐ and 5‐year survival times for high‐ and low‐risk patients. (F) Construction of a nomogram to predict the prognosis of kidney renal clear cell carcinoma (KIRC) patients. (G) Calibration curves of the column line graphs. (H) Demonstration of the nomogram and the net benefit of different characteristics.

Considering the robust correlation observed between the established risk model and unfavourable prognosis, we proceeded to perform univariate and multivariate independent prognostic analyses. These analyses aimed to ascertain the potential of the 20 genes to function as independent prognostic factors for KIRC patients. Univariate analyses revealed significant associations between Age (*p* < 0.001), Stage (*p* < 0.001), Grade (*p* < 0.001) and riskScore (*p* < 0.001) with KIRC patient prognosis (Figure 9A). Multivariate Cox analysis further confirmed a significant prognostic correlation for Stage, Age and riskScore (Figure 9B, *p* < 0.001), establishing riskScore as a reliable and independent predictor. The heatmap depicting the expression of 20 ITSGs in high‐ and low‐risk groups revealed that ARL4C, CLEC2B, CORO1B, PDCD1, PMAIP1, PPIB, RGS2, RPL13 and TNFRSF18 were highly expressed in the high‐risk group, indicating high‐risk genes. Conversely, the remaining 11 ITSGs were highly expressed in the low‐risk group, signifying protective genes. The heatmap further depicted the relationship between high‐risk and low‐risk groups and clinical traits, elucidating distinctions in the distribution of patients with varied clinicopathologic characteristics between the two groups (Figure 9C). Comparing risk scores with other clinical traits, the area under the ROC curve for the risk score was greater than that for all other clinical traits (Figure 9D). The ROC curves for 1‐, 3‐ and 5‐year survival predictions based on the risk score exhibited areas under the curve (AUC) values of 0.771, 0.748 and 0.772, respectively. These results underscore the heightened accuracy of the risk score in predicting patient survival when juxtaposed with alternative characteristics (Figure 9E). To enhance the clinical application and usability of the risk model, bar line graphs were created based on Gender, Grade, Age, Stage and Risk (Figure 8F). Calibration curves exhibited satisfactory concordance between predicted and observed values concerning 1‐, 3‐ and 5‐year overall survival (OS) probabilities. This observation attests to the stability of the nomogram plot (Figure 9G). The concordance index (c‐index) values pertaining to the nomogram net benefit consistently surpassed those associated with other clinical features, emphasizing the heightened accuracy of the constructed model in predicting patient survival (Figure 9H).

### 3.9 Differences in enrichment analysis in high‐ and low‐risk groups

**FIGURE 10:**
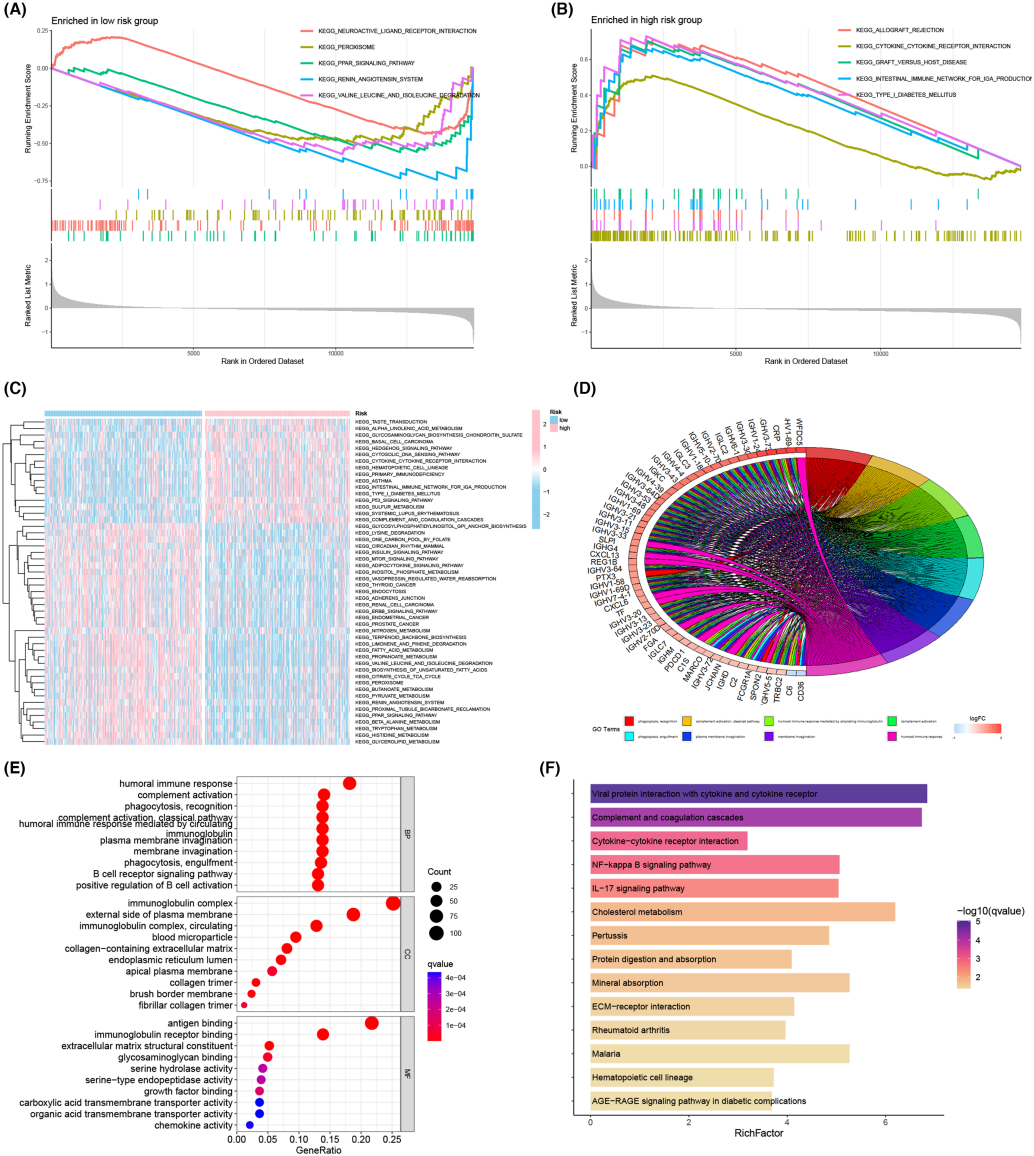
Enrichment analysis. (A, B) GSEA analysis of enrichment for low‐ and high‐risk groups separately. (C) GSVA analysis for high‐ and low‐risk groups. (D) Gene Ontology (GO) enrichment circle diagram. (E) Map of the top 10 GO‐enriched most enriched bubbles. (F) Top 13 Kyoto Encyclopedia of Genes and Genomes (KEGG)‐enriched most enriched bar graphs.

To understand the biological behaviours regulated by the identified ITSGs and their impact on patient prognosis, we conducted GSEA. Surprisingly, the low‐risk group exhibited upregulation in the pathway of neuroactive ligand‐receptor interactions, while downregulation was observed in pathways related to peroxisomes, PPAR signalling, renin‐angiotensin system, valine and isoleucine degradation. Conversely, the high‐risk group exhibited upregulation in pathways linked to allograft rejection, cytokine‐cytokine receptor interaction, graft versus host disease, intestinal immune network for IgA production and type I diabetes mellitus (Figure 10A,B). For a more detailed pathway enrichment analysis, GSVA revealed that taste transduction, α‐linolenic acid metabolism, chondroitin sulphate glycosaminoglycan biosynthesis, basal cell carcinoma, cell membrane DNA sensing pathway, haematopoietic cell lineage, primary immunodeficiency, psoriasis, intestinal immune network promoting IgA production, type I diabetes, p53 signalling pathway, sulphur metabolism, systemic lupus erythematosus, complement and coagulation cascade were highly expressed in the low‐risk group. Instead, the remaining KEGG pathways, with special attention to the KIRC pathway, were highly expressed in the high‐risk group, showing a significant correlation with poor prognosis in this group (Figure 10C). GO enrichment analysis revealed that in the low‐risk group, phagocytosis, recognition, complement activation, humoural immune response mediated by circulating immunoglobulins, plasma membrane invasion, membrane invagination and humoural immune response were highly expressed. The bubble diagrams further highlighted the close relationship between ITSGs and aspects of humoural immune response, immunoglobulin complexes and antigen binding (Figure 10D,E). KEGG results demonstrated that ITSGs were mainly enriched in pathways such as viral protein interaction with cytokines and cytokine receptors, complement and coagulation cascades, cytokine interaction with cytokine receptors, NF‐kappa B signalling pathway, IL‐17 signalling pathway and more (Figure 10F).

### 3.10 Assessment of immune infiltration

**FIGURE 11:**
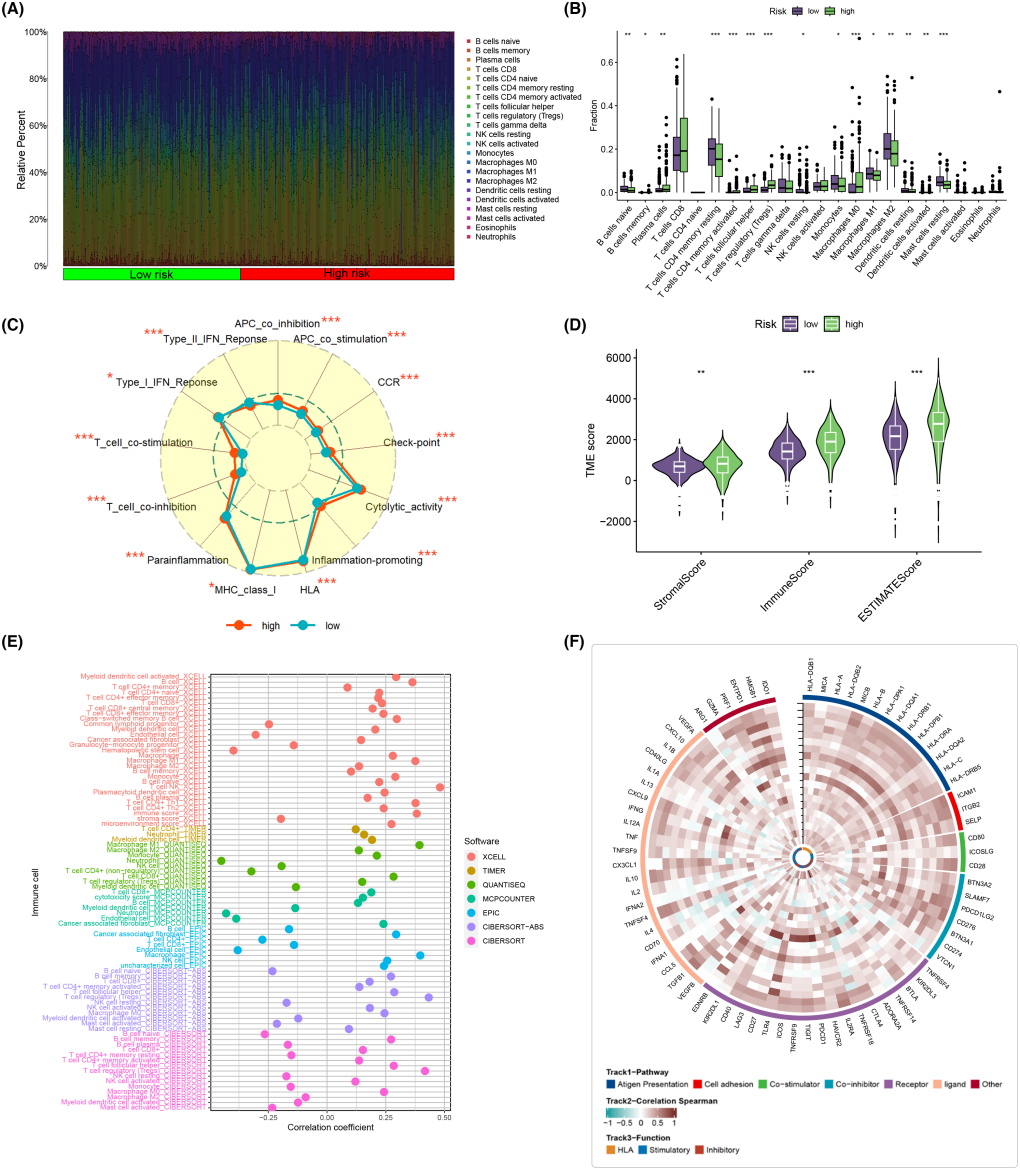
Immune infiltration assessment. (A) Percentage of each immune cell component in the high‐ and low‐risk groups. (B) Box plots comparing differences in immune cell expression in the high‐ and low‐risk groups. (C) Radar chart showing differences in immune function between high‐ and low‐risk groups. (D) Comparison of differences in three tumour microenvironment (TME) scores in high‐ and low‐risk groups. (E) Bubble plots of immune cell correlations under seven immune algorithms. (F) Heatmap of correlation between ITSGs and immune genes. *p < 0.05; **p < 0.01; ***p < 0.001.

To investigate the distribution of immune cells in the high‐ and low‐risk groups, we calculated the relative percentages of immune cells in each group (Figure 11A). The two groups exhibited distinct immune responses, with CD4 memory‐resting T cells being highly expressed in the low‐risk group, suggesting a potentially beneficial role in inhibiting tumour growth. Conversely, CD4 memory‐activated T cells, follicular helper T cells and regulatory T cells (Tregs) were highly expressed in the high‐risk group, indicating a more activated state of the immune system, possibly attempting to combat cancer development (Figure 11B). A comparison of immune functions between the high‐ and low‐risk groups using radar plots revealed that APC_co_inhibition, APC_co_stimulation, CCR, Check‐point, Cytolytic_activity, HLA, Inflammation‐promoting, MHC_class_I, Parainflammation, T_cell_co_inhibition, T_cell_co_stimulation and Type_I_IFN_Response were highly expressed in the high‐risk group. Conversely, Type_II_IFN_Response was highly expressed in the low‐risk group, indicating a more intense immune response in the high‐risk group, consistent with its poor prognosis (Figure 11C). Spearman's correlation analysis demonstrated a positive relationship between risk scores and immune infiltration, with the high‐risk group showing higher immune scores (Figure 11D). Further exploration of the correlation between risk scores and infiltrating immune cells using seven different immunization algorithms revealed that most immune cell infiltrates were positively correlated with risk scores, particularly T‐cell CD4+, T‐cell CD8+, T‐cell follicular helper and T‐cell regulatory (Tregs) (Figure 11E). To delve deeper into the relationship between ITSGs and immunity, we calculated the correlation between the 20 ITSGs and 75 key genes associated with immunity, considering immunity‐related pathways such as antigen presentation, cell adhesion, co‐stimulators, co‐inhibitors, receptors and ligands (Figure 11F).

### 3.11 ITSGs and immune checkpoint correlation analysis and TIDE analysis

**FIGURE 12:**
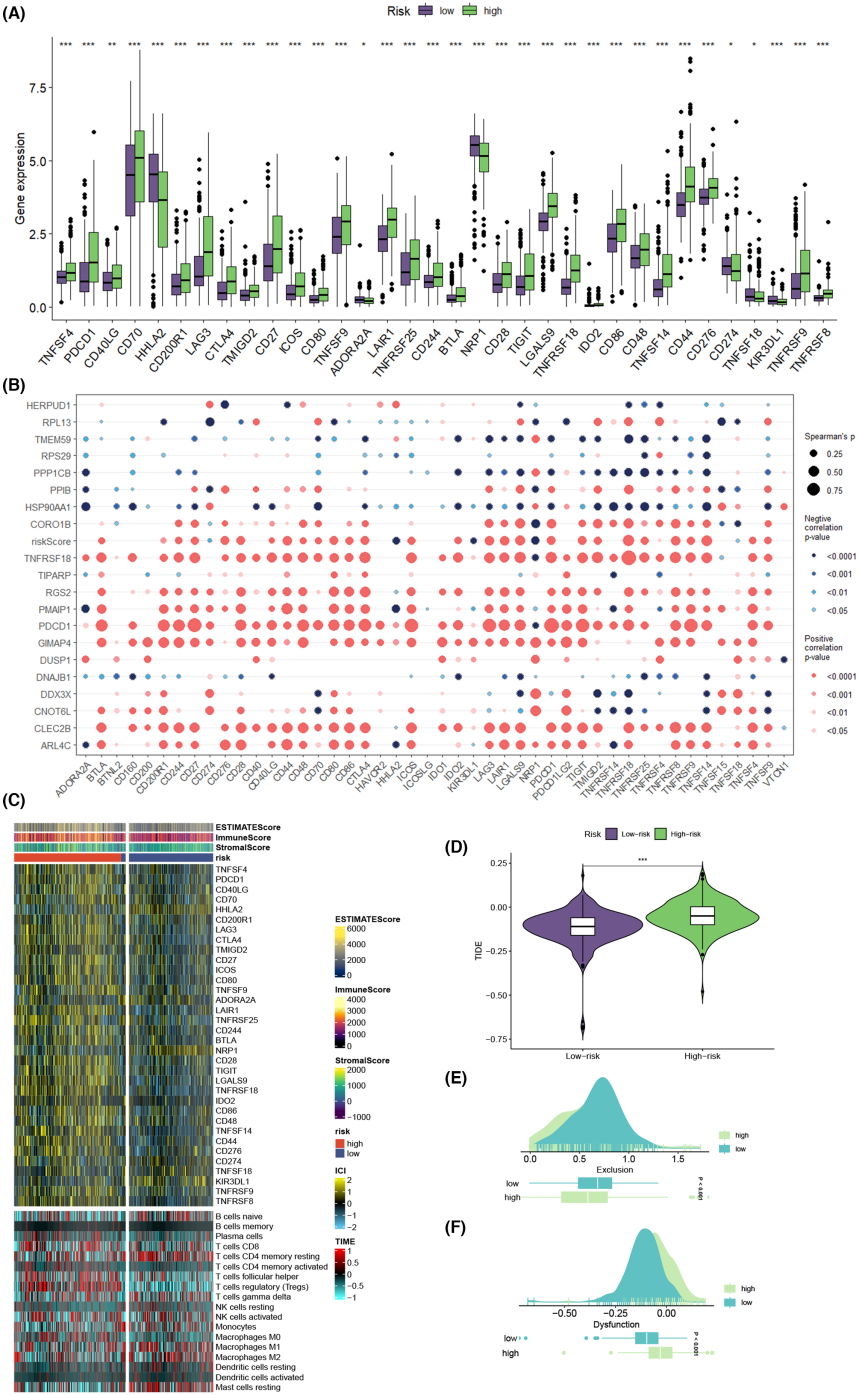
Immune checkpoint vs. tumour immune dysfunction and exclusion (TIDE) analysis. (A) Box plot of the difference in expression of immune checkpoint genes in high‐ and low‐risk groups. (B) Bubble plot of correlation between immune checkpoint genes and ITSGs, risk scores. (C) Heatmap integrating the relationship between immune checkpoint gene expression, immune cell abundance, immune microenvironment score and risk score. (D) Comparison of TIDE scores in high‐ and low‐risk groups. (E, F) Peak plots comparing Exclusion score with Dysfunction score.

In the realm of cancer treatment, immune checkpoint blockade therapy has become a common approach. We delved into the relationship between immune checkpoints and risk scores, discovering that the majority of immune checkpoint genes (ICGs), including TNFSF4, CD40LG and CD70, were highly expressed in the KIRC high‐risk group. Conversely, a small subset of immune checkpoint genes, such as HHLA2, ADORA2A, NRP1, TNFSF18 and KIR3DL1, exhibited higher expression in the KIRC low‐risk group (Figure 12A). Bubble plots were utilized to illustrate the correlation between ITSGs, risk scores and ICGs. Blue bubbles denoted negative correlation, while orange bubbles indicated positive correlation (Figure 12B). Intriguingly, most ITSGs exhibited positive correlation with immune checkpoints, with only HERPUD1, RPL13, TMEM59, RPS29, PPP1CB, PPIB and HSP90AA1 showing negative correlation. The risk score, instead, displayed positive correlation with most common immune checkpoints and negative correlation with a subset of immune checkpoints such as CD274, HHLA2, KIR3DL1 and NRP1. These insights offer new perspectives for potential immune checkpoint blockade therapies for KIRC patients. Furthermore, we presented the relationship between immune checkpoint gene expression, immune cell abundance, immune microenvironment score and risk score comprehensively in the form of a heatmap (Figure 12C). Subsequently, the comparison of TIDE scores between the high‐risk and low‐risk groups was conducted to scrutinize disparities in immune status. The findings revealed elevated TIDE scores within the high‐risk group (Figure 12D). The Exclusion score was higher in low‐risk patients, while the Dysfunction score exhibited the opposite trend (Figure 12E,F).

### 3.12 Exploring ITSGs in KIRC tumour mutations

**FIGURE 13:**
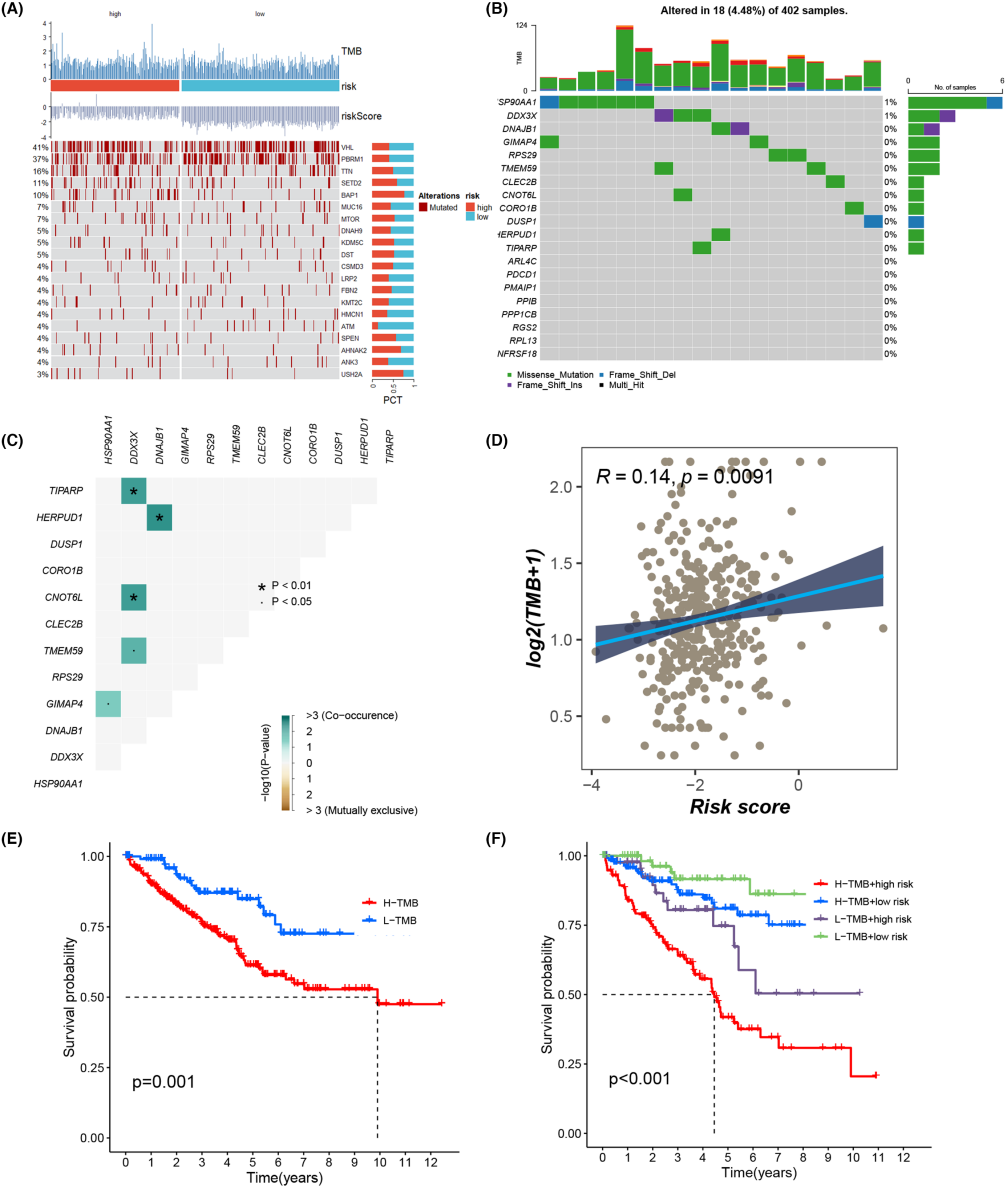
Tumour mutation profiles. (A) Heatmap of the relationship between high‐ and low‐risk groups with tumour mutation burden (TMB), risk scores and mutated genes. (B, C) Mutation profiles of 20 ITSGs in kidney renal clear cell carcinoma (KIRC). (D) Correlation between risk score and TMB. (E) Survival curve analysis of TMB in high‐ and low‐risk groups. (F) Survival curve analysis of TMB combined with risk score in high‐ and low‐risk groups. *p < 0.05.

Somatic mutations play a crucial role in shaping the outcomes of cancer immunotherapy. We delved into the mutation spectrum of TCGA‐KIRC, establishing connections between high‐ and low‐risk groups with TMB, risk scores and mutated genes (Figure 13A). Simultaneously, we explored the potential mutation of the 20 ITSGs in KIRC, identifying possibilities in HSP90AA1 and DDX3X (Figure 13B). Moreover, we unveiled co‐occurrence relationships among ITSGs in tumour mutations, such as TIPARP with DDX3X and HERPUD1 with DNAJB1 (Figure 13C). Spearman correlation analysis revealed a significant and positive correlation between TMB and risk score (Figure 13D, *R* = 0.14, *p* = 0.0091). To delve deeper into the relationship between high‐ and low‐mutation loads and patient survival time, we conducted survival curve analysis. The results indicated that KIRC patients with lower TMB had a better prognosis, while those with higher TMB, especially in conjunction with high‐risk scores, exhibited the worst prognosis (Figure 13E,F). This highlights the importance of tumour mutation load and risk score in determining the prognosis of KIRC patients.

### 3.13 Risk scores based on ITSGs to predict drug sensitivity for the treatment of KIRC

**FIGURE 14:**
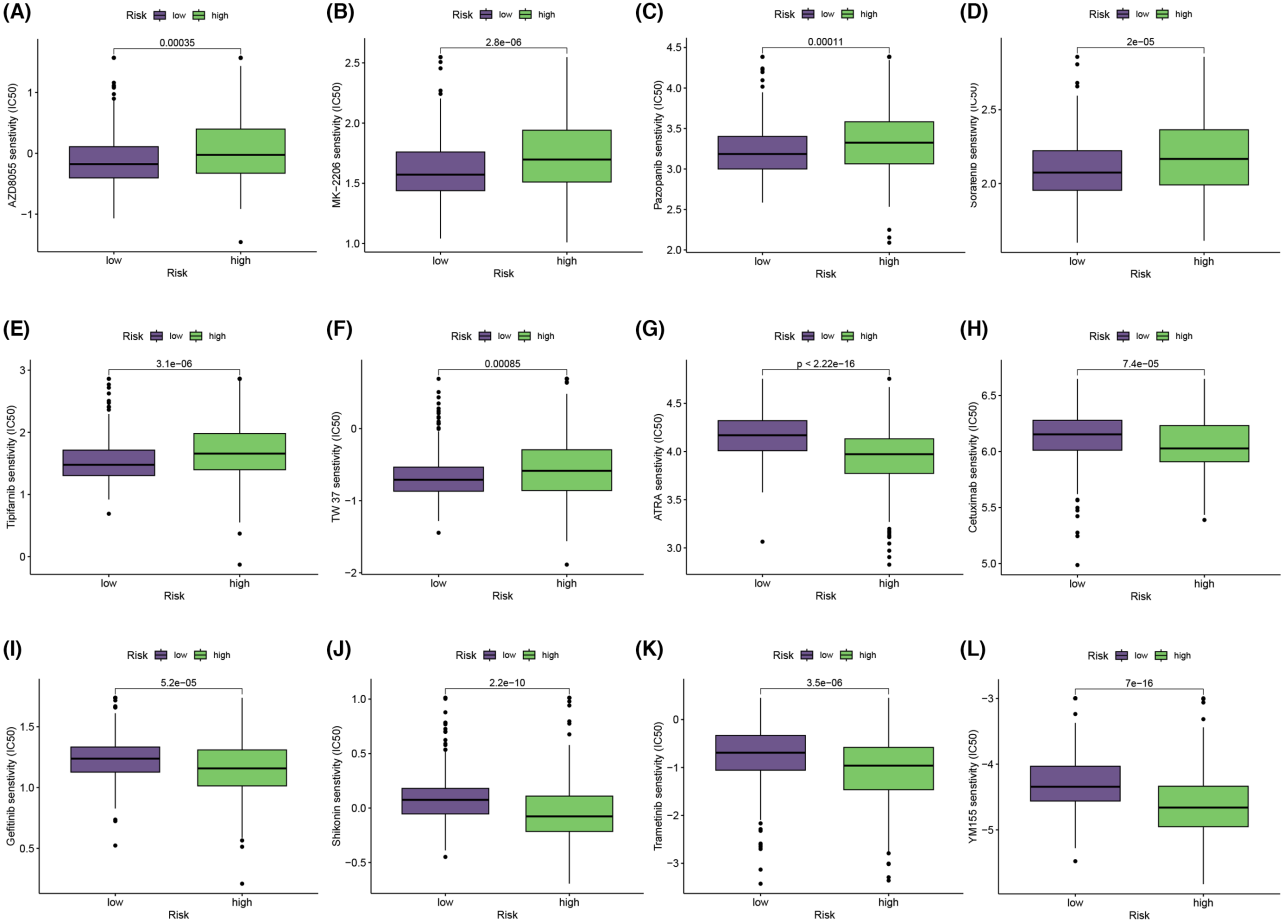
Differences in IC50 comparing different immunotherapy drugs: (A) AZD8055, (B) MK‐2206, (C) pazopanib, (D) sorafenib, (E) tipifarnib, (F) TW37, (G) ATRA, (H) cetuximab, (I) gefitinib. (J) shikonin, (K) trametinib, (L) YW155.

Leveraging risk scores derived from ITSGs enables a more nuanced analysis of immunotherapy efficacy in KIRC patients, aiding in the optimization of drug dosages. Our predictions for immunotherapeutic drugs in KIRC highlighted significant differences in sensitivity between high‐ and low‐risk groups for 12 immunologic drugs (*p* < 0.05). Six drugs (AZD8055, MK‐2206, pazopanib, sorafenib, tipifarnib and TW37) exhibited higher sensitivity in the low‐risk group, as indicated by lower IC50 values (Figure 14A–F). Conversely, the remaining six drugs (ATRA, cetuximab, gefitinib, shikonin, trametinib and YW155) demonstrated higher sensitivity in the high‐risk group, with larger IC50 values in the low‐risk group (Figure 14G–L). These findings provide valuable insights into the immunotherapy response of KIRC patients and offer avenues for refining precision in drug.

### 3.14 The role of PPIB in fostering the proliferation, invasion and migration of KIRC has been uncovered through meticulous in vitro investigations

**FIGURE 15:**
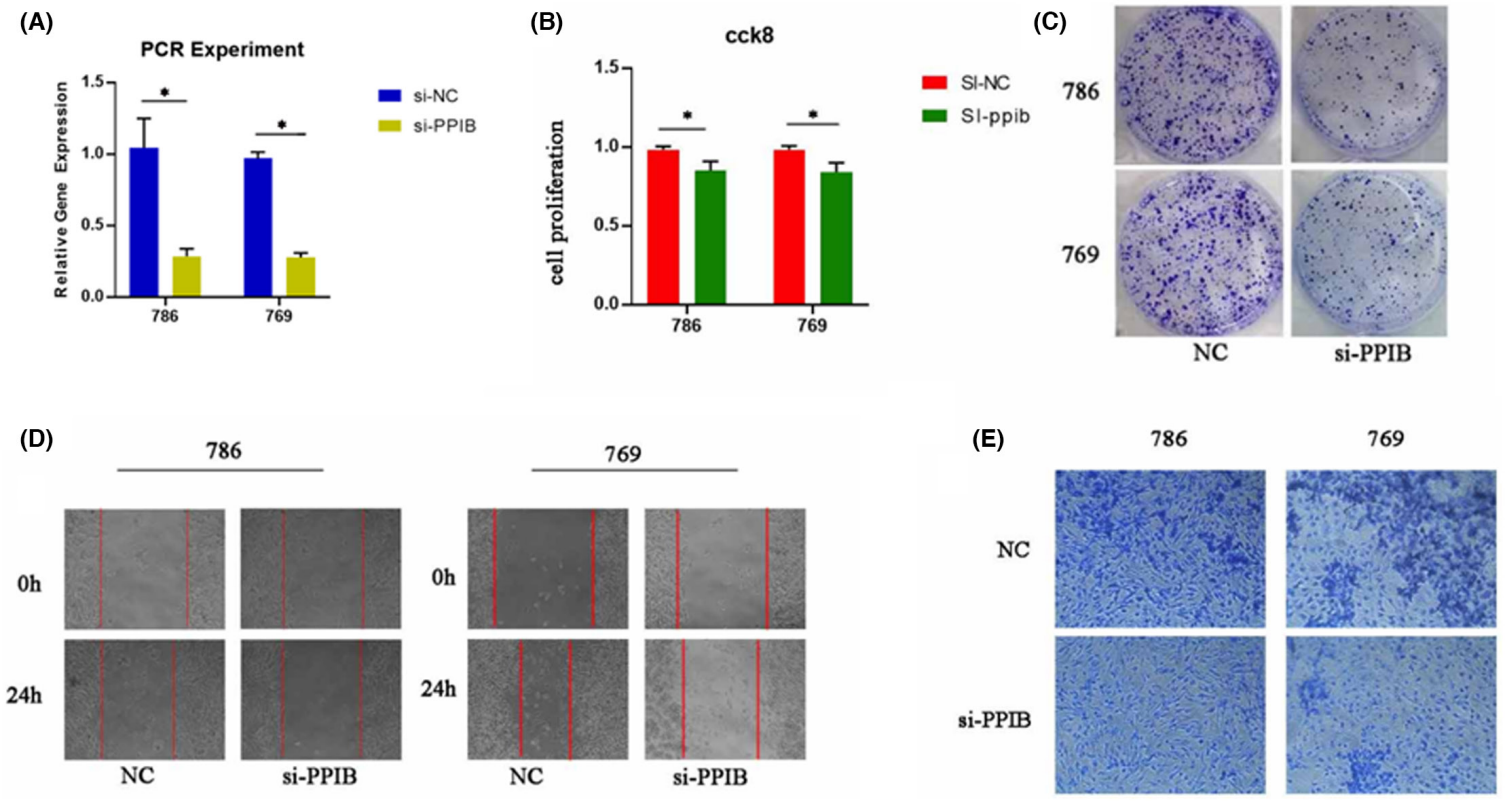
Experimental investigation of the role of PPIB in tumour proliferation, invasion and migration in kidney renal clear cell carcinoma (KIRC). (A) PCR experiments in two KIRC cells, 786 and 769 (B) CCK‐8 analysis, (C) plate cloning assay, (D) wound‐healing assay and (E) Comparison of Transwell assays performed in two cell chambers. *p < 0.05.

Employing a battery of experimental approaches, we sought to elucidate the potential implications of PPIB in KIRC pathogenesis. In the PCR experiment, the expression of PPIB in KIRC cells underwent significant downregulation after it was knocked down (Figure 15A). Notably, utilizing the CCK‐8 assay, we observed a pronounced diminution in the proliferation rate of KIRC cells subsequent to PPIB silencing, as evidenced by Figure 15B. Furthermore, plate cloning assays revealed a marked decrease in tumour cell abundance upon PPIB knockdown, signifying a dampened proliferative and invasive propensity compared with control conditions (Figure 15C). In parallel, wound‐healing assays showed a significant decrease in the migration ability of tumour cells with the downregulation of PPIB expression levels (Figure 15D). Complementary Transwell assays further corroborated these observations, demonstrating a significant impediment in KIRC cell invasion and migration following interference with PPIB expression (Figure 15E). Collectively, these findings underscore the pivotal role of PPIB in driving tumorigenesis, wherein its upregulation substantiates enhanced proliferation, invasion and migration capabilities within KIRC cells.

## 4 DISCUSSION

Renal clear cell carcinoma (RCC), a prevailing subtype of renal cell carcinoma, stands as a formidable and highly fatal malignancy, imposing a substantial burden on societal healthcare systems. Precision in predicting its progression and enhancing therapeutic strategies remains a paramount research focus.^37^, ^38^ Typically characterized by asymptomatic or nonspecific symptoms, RCC often eludes early detection, adversely impacting patient prognoses.^39^ While targeted therapeutic agents, including sunitinib, sorafenib, bevacizumab and tesilomox, have been pivotal in RCC treatment, the efficacy of anti‐angiogenic and targeted therapies requires refinement, with surgical intervention retaining its primary role.^40^, ^41^, ^42^ The quest for novel therapeutic and prognostic models remains imperative. However, the diverse treatment responses and disease progressions observed in RCC underscore the intricate interplay of individual variations, potentially linked to distinct epigenetic profiles, the tumour microenvironment and heightened heterogeneity.^43^, ^44^

Traditional tumour staging, focusing solely on static tumour status at a specific time point, lacks the ability to capture dynamic cellular dynamics, the tumour microenvironment and immune characteristics. Consequently, it falls short in accurately predicting disease progression and treatment responses for patients.^23^, ^45^, ^46^ The integration of bioinformatics has emerged as a pivotal avenue in KIRC research. Recent investigations underscore the crucial role of T cells in RCC, particularly emphasizing their association with prognosis.^47^ Notably, tumour‐infiltrating T cells, especially within the tumour epithelium, correlate strongly with favourable outcomes. This association is particularly pronounced for the CD8+ T‐cell subset, highlighting the pivotal role of cytotoxic T‐lymphocytes (CTL) in antitumour immune responses.^48^, ^49^ Our study aims to construct a robust prognostic model for RCC, leveraging T‐cell signature genes through single‐cell histology and transcriptomics. This model holds promise in enhancing prognostic insights, elucidating mechanisms of tumour progression and laying the groundwork for innovative therapeutic strategies, encompassing immunotherapy, targeted molecular interventions and personalized treatment approaches.

We curated raw KIRC data from diverse repositories, including the GEO database, the Xena database and the Arrayexpress database, encompassing single‐cell RNA sequencing data and transcriptome data. Initially, we subjected the single‐cell RNA sequencing data to comprehensive analyses involving dimensionality reduction, clustering, cellular communication analysis and proposed time‐series analysis. Subsequent to this, we employed the ‘hdWGCNA’ R package to execute high‐dimensional weighted gene co‐expression network analysis, facilitating the identification of core genes associated with 150 pivotal T‐cell subpopulations. Leveraging one‐way Cox regression and machine learning, we identified immune‐related genes with prognostic significance. Comprising 10 diverse machine learning algorithms, our combinatorial analysis selected the optimal algorithm based on the C‐index values, filtering the most relevant ITSGs for constructing the prognostic model.^50^ For enhanced clinical management and prognostic assessment in KIRC patients, we developed Nomogram, calibration curves and decision curves.^51^ Additionally, we conducted multiple enrichment analyses to unravel the functional roles and pathways governed by these ITSGs. A meticulous exploration of immune infiltration correlation shed light on the intricate interplay between KIRC and the immune system. Employing the R package ‘maftools’, we conducted a detailed analysis of tumour gene mutations. Furthermore, drug sensitivity analysis identified potentially effective drugs, offering valuable insights for KIRC treatment. Finally, to validate our findings, we performed qPCR, CCK‐8 and Transwell assays.

Cells intricately regulate metabolic, proliferative and intercellular communication processes through signalling pathways, responding to environmental cues.^52^ This signalling network incorporates transcription factors and cytokines within distinct cellular contexts, exerting pivotal roles in pathway regulation.^53^, ^54^ Among these, macrophage migration inhibitory factor (MIF) emerges as a cytokine with diverse functions. MIF not only activates T‐lymphocytes but also impedes macrophage migration.^55^ Its involvement extends across immune regulation, inflammation, tumour development and metabolic control.^56^ Functioning as a pro‐inflammatory cytokine, MIF, primarily sourced from T cells, induces the production of inflammatory mediators, including TNF‐alpha, IL‐1 beta, IL‐6 and IL‐8.^57^, ^58^ Extracellularly secreted MIF binds to receptors, notably CD74, initiating a cascade of signalling pathways.^59^, ^60^ In KIRC, MIF assumes a dual role, exerting its significance. In immunomodulation, MIF activates immune cells, particularly T cells, prompting the release of pro‐inflammatory factors and triggering an immune response.^61^ Simultaneously, MIF inhibits the effects of anti‐inflammatory cytokines, such as glucocorticoids, contributing to inflammation and immune response maintenance.^62^ In the context of cancer, MIF is intricately linked to the development and progression of KIRC. As a tumour‐secreted factor,^57^ MIF not only directly promotes tumour cell proliferation, invasion and metastasis^63^ but also remodels the tumour microenvironment by modulating inflammatory cell and haematopoietic stem cell recruitment, thereby supporting tumour growth and survival.^64^, ^65^ This multifaceted role emphasizes the complex interplay of MIF with T cells in KIRC, impacting diverse biological processes such as T‐cell immunomodulation, inflammation and tumour development. Recognizing its potential as a therapeutic target,^57^, ^66^ MIF emerges as a key player in the intricate landscape of KIRC.

In this study, the investigation of ITSGs has highlighted the significance of specific genes in patients with KIRC. Notably, PDCD1, responsible for encoding the PD‐1 protein, emerges as a focal point. PD‐1, expressed on both CD4+ and CD8+ T cells, is implicated in T‐cell activity and heterogeneity within KIRC patients.^67^, ^68^ Operating as a T‐cell immune checkpoint receptor, PD‐1 inhibits T‐cell activity by binding to its ligands PD‐L1 or PD‐L2.^69^ In KIRC, the tumour cells commonly overexpress PD‐L1, leading to immune evasion and the suppression of T‐cell function, ultimately reducing their immune response against the carcinoma.^70^ Targeting the PD‐1/PD‐L1 pathway provides a strategy to activate T cells and enhance their immune response to KIRC. However, it is crucial to recognize that PD‐1 may exhibit heterogeneity among patients and different T‐cell subsets, influencing its response to therapy.^71^ Another prominent ITSG, TNFRSF18, encodes GITR, a tumour necrosis factor receptor family member pivotal in T‐cell regulation and activity.^72^ GITR engagement augments immunity by delivering costimulatory signals that enhance T‐cell proliferation and effector function while mitigating the immunosuppressive effects of Tregs.^73^ The expression of GITR and its ligand, GITRL, appears to play a role in modulating T‐cell function and antitumour immunity in patients with KIRC.^74^ GITR, a receptor on T‐cell surfaces, activates a signalling pathway upon binding to its ligand, GITRL. This interaction, whether through GITRL or agonist antibody to GITR, enhances T‐cell activation by inducing the expression of IL‐2 and IFN‐γ, augmenting CD25 expression and promoting cell expansion.^75^ This signifies an increased activity of T cells, resulting in a more robust immune response. Certainly, the collaborative effects of diverse immunotherapeutic strategies often lead to more robust immune responses, offering a more potent defence against malignancies like KIRC.^76^ The activation of GITR, a key player identified in this study, could synergize effectively with other immunotherapeutic approaches, including anti‐PD‐1/PD‐L1 therapy.^77^ This combined treatment strategy holds the potential to evoke a heightened immune response, presenting a more formidable defence against KIRC. Such findings pave the way for innovative individualized treatment approaches aimed at enhancing therapeutic efficacy and ultimately improving patient prognosis.

The relationship of gene PPIB with KIRC and T cells has been characterized in studies. The protein encoded by PPIB is an isomerase that catalyses protein folding and participates in the process of protein synthesis and modification.^78^ PPIB may influence T‐cell function and activity, which in turn affects the immune response. In addition, PPIB may interact with other immunomodulatory factors in the tumour microenvironment to influence T‐cell infiltration and activity.^78^, ^79^ Understanding the expression of PPIB in KIRC patients and its relationship with T cells can help to gain a deeper understanding of the immunoregulatory mechanisms of KIRC and provide new clues for the development of individualized treatment strategies.

CD4 memory‐quiescent T cells represent a subset of immune memory cells that previously acquired memory during the immune system's response to pathogens or foreign entities, exhibiting an enduring half‐life and currently residing in a quiescent state. Renowned for their immunomodulatory potential, these cells engage in isotypic interactions with activated T cells, prompting the production of cytokines (IL‐10, IL‐4, IL‐7) with immunomodulatory properties.^80^ This cytokine release aids in balancing the immune response, preventing excessive inflammation and mitigating autoimmune diseases.^81^, ^82^ Notably, CD4+ T memory cells play a pivotal role in bolstering anti‐tumour responses. They achieve this by enhancing clonal expansion at the tumour site, preventing activation‐induced cell death and preferentially fostering the generation of immune memory cells from CTLs acting as antigen‐presenting cells.^83^, ^84^ In the context of KIRC, the heightened expression of CD4 memory‐resting T cells assumes a protective role, contributing to the maintenance of immune homeostasis and the suppression of over‐activated immune responses.^85^, ^86^ This inhibitory effect serves to attenuate chronic inflammation within the TME, thereby reducing factors that promote tumour growth.^87^ Additionally, CD4 memory‐quiescent T cells play a crucial role in defending against tumours by regulating the activity of other immune cells, such as CD8+ T cells and natural killer cells.^88^ In summary, the elevated expression of CD4 memory‐resting T cells in the low‐risk group suggests a well‐regulated immune system, potentially contributing to the effective response against KIRC. Notably, a robust association between T cells and epithelial cells emerged upon investigating spatial cellular interplays within the tumour microenvironment. Remarkably, even at a distance, T cells persist in their interactions with epithelial counterparts. This apparent correlation between T cells and epithelial cells implies a strong antitumour effect of T cells, especially in the case of immune cells infiltrating tumour tissue.

Immune checkpoints serve as intrinsic regulatory mechanisms essential for maintaining immune system balance.^89^ Immune checkpoint therapy, involving the blockade or activation of specific immune checkpoint molecules, has the potential to fortify or reactivate the immune system, enhancing its efficacy in combating tumour cells.^90^, ^91^ Notably, in KIRC, CD70 assumes a pivotal role as an immune checkpoint, with its expression levels exhibiting significant differences between high‐risk and low‐risk groups.^92^ CD70 is highly expressed in the high‐risk group and relatively low in the low‐risk group, suggesting a potential correlation between CD70 expression, disease severity and prognosis in KIRC patients.^93^ CD70, typically expressed by tumour cells, serves as a receptor‐ligand binding to the CD27 receptor on T cells' surface.^94^ This interaction activates the CD27/CD70 signalling pathway, promoting effector and memory T‐cell differentiation in CD8+ T cells, thereby enhancing their functionality and active participation in the immune response against tumours.^95^, ^96^ CD70's effects encompass heightened T‐cell cytotoxicity, induced cytokine production and improved T‐cell viability^97^ Elevated CD70 expression may stimulate a more robust anti‐tumour immune response, restraining tumour growth. Conversely, diminished CD70 expression could be associated with suppressed immune function, contributing to tumour progression. This insight lays the groundwork for developing immunotherapeutic strategies aimed at modulating CD70 expression^98^ to enhance T‐cell immune activity, ultimately improving therapeutic efficacy and prognosis for KIRC patients.

Additionally, the TIDE tool, assessing tumour immune function, aids in comprehending the intricate interplay between immune response and therapeutic outcomes in tumour patients.^99^, ^100^ TIDE operates through two crucial dimensions: immune dysfunction and immune exclusion.^101^ Tumour cells employ diverse mechanisms to elude immune detection and attack, including diminished antigen expression, mutations and surface protein variations. These tactics pose challenges for the immune system in recognizing and combating tumour cells.^102^, ^103^, ^104^ A heightened TIDE score may signal the presence of immunosuppressive factors, such as immunosuppressive cytokines or an upsurge in suppressor T cells (Tregs).^105^ These elements can impede immune cell function, diminishing their potency against tumours. An elevated TIDE score may indicate an insufficiency of activated immune cells, encompassing CD8+ T cells, natural killer cells (NK cells), etc., in the tumour vicinity,^106^ resulting in an inadequate local immune response. Elevated TIDE scores point towards intricate mechanisms of immunosuppression and exclusion, potentially limiting the efficacy of immunotherapy. Thus, delving into the intricacies of TIDE's mechanism provides a comprehensive understanding of the tumour immune environment's characteristics, paving the way for the development of more effective immunotherapy strategies.

The predictive efficacy of our prognostic model, anchored in ITSGs, proves substantial for KIRC patient prognosis. However, acknowledging inherent limitations in our study is imperative. A primary constraint arises from our exclusive reliance on publicly available datasets from repositories like GEO, Xena and Arrayexpress, introducing a potential real‐world bias and requiring cautious interpretation of our findings. Despite efforts to address this limitation by exploring alternative resources, the scarcity of available KIRC data compelled us to opt for experimental validation. To enhance the model's accuracy, comprehensive clinical data from a sizable KIRC patient cohort, including details on their engagement in immunotherapeutic interventions, is essential. This augmented dataset will serve as a reference standard, fortifying the model's empirical validity and affirming its accuracy and applicability. In summary, while our investigation underscores the considerable promise of our prognostic model, it underscores the importance of acknowledging and mitigating constraints linked to data accessibility and experimental validation. Collaborating with more extensive clinical cohorts and incorporating detailed treatment data will significantly bolster the credibility and utility of our model.

## 5 CONCLUSION

In summary, our investigation systematically delineated the heterogeneity inherent within infiltrating T cells in KIRC, leveraging both single‐cell RNA sequencing data and spatial transcriptome data. Concurrently, through the employment of a multi‐omics integration algorithm, we successfully discerned ITSGs and devised a robust prognostic model, thereby unearthing the prognostic significance of infiltrating T cells in KIRC. Furthermore, employing the RCTD, we elucidated the spatial interactions among diverse cell types within KIRC spatial transcriptome sections. Our observations also shed light on the immune microenvironment prevailing in KIRC and appraised the variable responses elicited by various chemotherapeutic agents, thus furnishing prospective avenues for clinicians to tailor personalized treatment modalities and immunotherapies.

## AUTHOR CONTRIBUTIONS

**Hao Chi:** Conceptualization (equal); writing – review and editing (equal). **Haiqing Chen:** Conceptualization (equal); data curation (equal); formal analysis (equal); validation (equal); visualization (equal); writing – original draft (equal). **Haoyuan Zuo:** Data curation (equal); formal analysis (equal); visualization (equal); writing – original draft (equal). **Jinbang Huang:** Data curation (equal); formal analysis (equal); visualization (equal); writing – original draft (equal). **Jie Liu:** Data curation (equal); formal analysis (equal); visualization (equal); writing – original draft (equal). **Lai Jiang:** Formal analysis (equal); visualization (equal); writing – original draft (equal). **Chenglu Jiang:** Validation (equal); writing – original draft (equal). **Shengke Zhang:** Validation (equal); writing – original draft (equal). **Qingwen Hu:** Writing – original draft (equal). **Haotian Lai:** Writing – original draft (equal). **Bangchao Yin:** Writing – original draft (equal). **Guanhu Yang:** Writing – review and editing (equal). **Gang Mai:** Conceptualization (equal); writing – review and editing (equal). **Bo Li:** Conceptualization (equal); resources (equal); writing – review and editing (equal).

## FUNDING INFORMATION

The study was approved by the Dazhou Science and Technology Bureau project (21ZDYF0025), the Sichuan Medical Association Project (S21048) and the Sichuan Provincial Science and Technology Department Project (22ZDYF1898).

## CONFLICT OF INTEREST STATEMENT

The authors declare that they conducted this study free from any current or potential commercial or financial affiliations that could create a conflict of interest.

## Supporting information


Table S1

## Data Availability

The datasets utilized in our investigation are accessible via the GEO repository (https://www.ncbi.nlm.nih.gov/geo/) and the Xena database (https://xenabrowser.net/hub/) and the Arrayexpress database (https://www.ebi.ac.uk/biostudies/arrayexpress/).
